# Extracellular vesicles produced by avian pathogenic *Escherichia coli* (APEC) activate macrophage proinflammatory response and neutrophil extracellular trap (NET) formation through TLR4 signaling

**DOI:** 10.1186/s12934-023-02171-6

**Published:** 2023-09-09

**Authors:** Zhongxing Wang, Dongyu Zhu, Yuting Zhang, Fufang Xia, Jiaying Zhu, Jianjun Dai, Xiangkai Zhuge

**Affiliations:** 1 Key Lab of Animal Bacteriology, MOE Joint International Research Laboratory of Animal Health and Food Safety, College of Veterinary Medicine, Ministry of Agriculture, Nanjing Agricultural University, No.1 Weigang road, Nanjing, 210095 China; 2 Department of Nutrition and Food Hygiene, School of Public Health, Nantong University, No.9 Seyuan road, Nantong, Jiangsu 226019 P.R. China; 3 College of Pharmacy, China Pharmaceutical University, Nanjing, 211198 China

**Keywords:** Avian pathogenic *Escherichia Coli*, Extracellular vesicle, TLR4 signaling, Inflammatory response, Neutrophil extracellular trap

## Abstract

**Background:**

Avian pathogenic *Escherichia coli* (APEC) is the major pathogen causing important avian diseases in poultry. As an important subtype of extraintestinal pathogenic *E. coli*, APEC has zoonotic potential and is considered a foodborne pathogen. APEC extracellular vesicles (EVs) may play vital roles in the interaction of the pathogen with its host cells. However, the precise roles played by APEC EVs are still not completely clear, especially in immune cells.

**Results:**

In this study, we investigated the relationships between APEC EVs and immune cells. The production and characteristics of the EVs of APEC isolate CT265 were identified. Toll like receptor 4 (TLR4) triggered the cellular immune responses when it interacted with APEC EVs. APEC EVs induced a significant release of proinflammatory cytokines in THP-1 macrophages. APEC EVs induced the macrophage inflammatory response via the TLR4/MYD88/NF-κB signaling pathway, which participated in the activation of the APEC-EV-induced NLRP3 inflammasome. However, the loss of lipopolysaccharide (LPS) from APEC EVs reduced the activation of the NLRP3 inflammasome mediated by TLR4/MYD88/NF-κB signaling. Because APEC EVs activated the macrophage inflammatory response and cytokines release, we speculated that the interaction between APEC EVs and macrophages activated and promoted neutrophil migration during APEC extraintestinal infection. This study is the first to report that APEC EVs induce the formation of neutrophil extracellular traps (NETs) and chicken heterophil extracellular traps. Treatment with APEC EVs induced SAPK/JNK activation in neutrophils. The inhibition of TLR4 signaling suppressed APEC-EV-induced NET formation. However, although APEC EVs activated the immune response of macrophages and initiated NET formation, they also damaged macrophages, causing their apoptosis. The loss of LPS from APEC EVs did not prevent this process.

**Conclusion:**

APEC-derived EVs induced inflammatory responses in macrophages and NETs in neutrophils, and that TLR4 was involved in the APEC-EV-activated inflammatory response. These findings provided a basis for the further study of APEC pathogenesis.

**Supplementary Information:**

The online version contains supplementary material available at 10.1186/s12934-023-02171-6.

## Background

Avian pathogenic *Escherichia coli* (APEC) is the commonest pathogen of birds, and causes avian colibacillosis worldwide [1]. It is an important subtype of extraintestinal pathogenic *E. coli* (ExPEC), and causes a variety of local and systemic infections in poultry of different ages [2, 3]. Recent research indicates that APEC has zoonotic potential [4–6], and it is recognized as a foodborne pathogen. The avian diseases caused by APEC include perihepatitis, airsacculitis, pericarditis, and other syndromes, such as egg peritonitis, salpingitis, coligranuloma, omphalitis, and cellulitis, collectively known as “colibacillosis” [7]. APEC causes poultry disease via specific virulence factors, such as adhesins, invasins, protectins, iron acquisition systems, and toxins. These virulence factors promote APEC invasion and its evasion of the host immune responses, causing extraintestinal infections [8]. After invading the host, APEC colonizes the lungs, evades the body’s immune system, and enters the bloodstream circulation, subsequently causing systemic infections [9]. As a facultatively intracellular pathogen, APEC can survive in avian macrophages [10, 11], and its intracellular colonization is affected by many intracellular survival factors. The ColV plasmid plays key roles in the survival of APEC within macrophages. Our previous research showed that the two-component system PhoP/PhoQ regulates the expression of hemolysin HlyF (the encoding gene *hlyF* is located on the ColV plasmid) to promote the escape of APEC and ExPEC from macrophage phagolysosomes into the cytosol [12].

Bacteria can produce extracellular vesicles (EVs) during their normal growth [13]. Bacterial EVs are classified into many subtypes according to their different methods of production, including outer-membrane vesicles (OMVs), outer-inner membrane vesicles (OIMVs), explosive outer membrane vesicles (EOMVs), and cytoplasmic membrane vesicles (CMVs) [14], which are collectively referred to as “EVs”. Bacterial EVs are usually nanovesicles of 20–400 nm [15]. As accessory substances produced by bacteria, EVs carry specific subsets of bacterial molecular cargo, such as DNA, RNA, protein, etc., some of which play important roles in the information exchange between and within bacterial species [16, 17]. Like other *E. coli* strains, APEC and ExPEC also produce extracellular vesicles (EVs), and studies have shown the potential utility of EVs in gene delivery and vaccine development [18–21].

Recent studies have demonstrated that EVs played vital roles in the interactions between pathogens and host cells, and induce specific host responses. For example, *Legionella pneumophila*-derived OMVs promote bacterial replication in macrophages [22]. *Staphylococcus aureus* EVs activate innate immune receptors and induce autophagy [23]. OMVs protect Gram-negative bacteria against host defense peptides [24]. These reports demonstrate that EVs are the natural secretion products of bacteria, and that EVs produced by pathogens exert damaging effects by interacting with host cells. Although there have been many reports of APEC and their pathogenesis, there have been few reports of the role of APEC-derived EVs in APEC virulence during infection, especially regarding whether EVs interact with immune cells to change their physiological functions.

As the first line of the host defenses, the innate immune system plays a vital role in the development of many inflammatory diseases. The innate immune system includes tissue barriers, innate cellular immunity, and innate molecular immunity. Macrophages and neutrophils are essential parts of the host’s innate immune defenses against bacterial infections [25]. Macrophages are important phagocytic cells, phagocytizing pathogenic microorganisms. Moreover, they can present antigens and release cytokines that participate in the inflammatory response and pathogen clearance [26, 27]. In addition to their roles in the innate immune response, macrophages activate adaptive immune cells, especially T cells, to migrate to the sites of bacterial infection to kill infected cells [28]. After pathogenic microorganisms invade the body, neutrophils are rapidly activated, and migrate to the area of inflammation [29]. The three major mechanisms by which neutrophils kill microbes are phagocytosis, degranulation, and the release of neutrophil extracellular traps (NETs) [30], the most recently identified way by which they eliminate invasive pathogens [31]. NETs are composed of DNA, histones, and numerous granule proteins, including neutrophil elastase (NE) and myeloperoxidase (MPO) [32, 33]. Several studies have reported that EVs induce inflammatory responses in macrophages and neutrophils [34–36]. However, the effects of APEC-derived EVs on macrophages and neutrophils have not yet been investigated. In this study, we investigated the molecular mechanisms by which APEC-derived EVs induce an inflammatory response.

## Results

### Production and characterization of EVs from APEC wild-type isolate, CT265

**Fig. 1:**
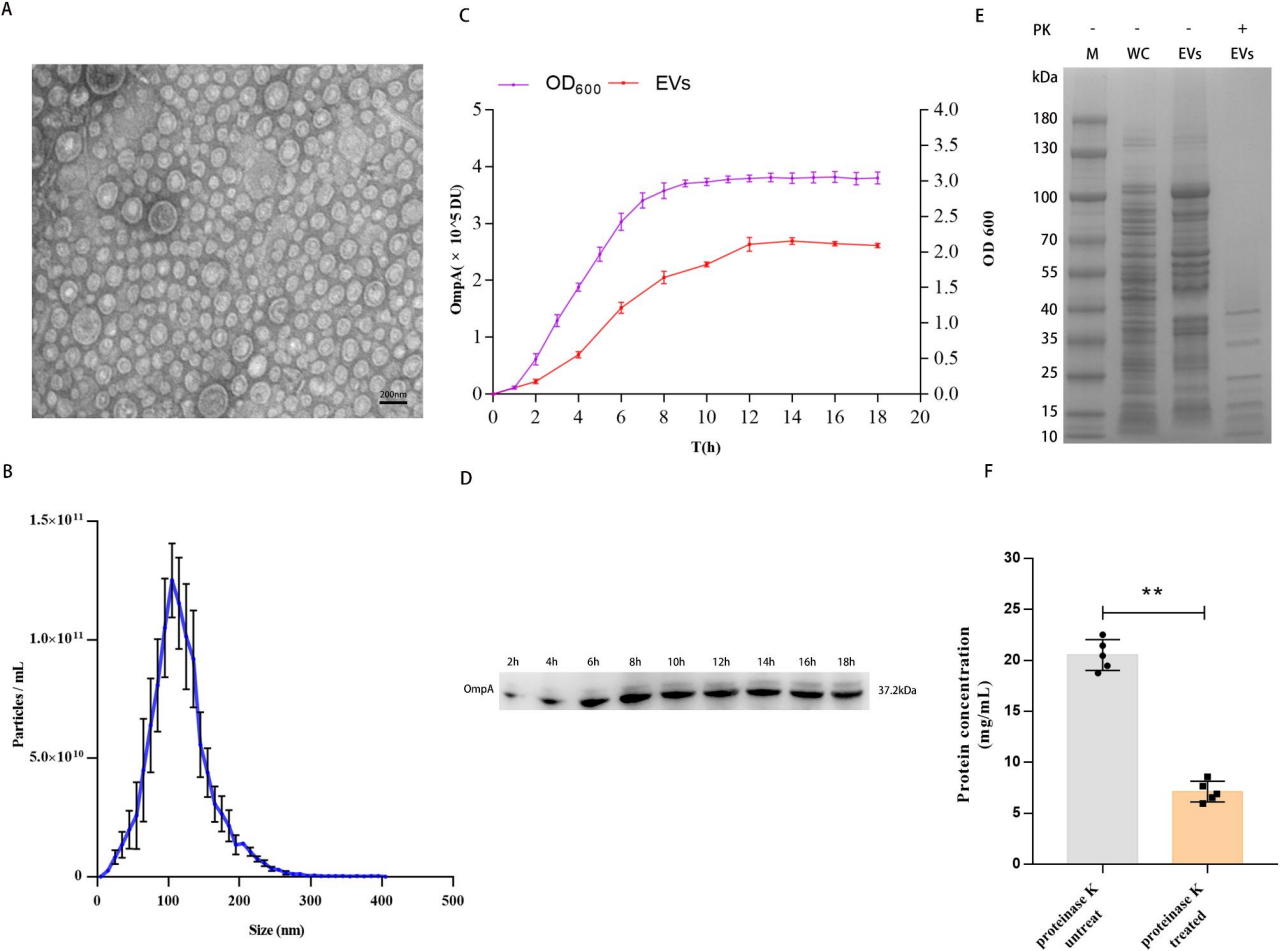
Characterization and kinetics of *E. coli* CT265 EVs. (**A**) Transmission electron microscopy (TEM) image of negatively stained APEC CT265 EVs (n = 1). Scale bars: 200 nm. (**B**) Size distribution of CT265 EVs determined with nanoparticle tracking analysis (NTA). Data are the means of three biological replicates (blue line) ± standard errors of the means. (**C**) Kinetic curves for *E. coli* CT265 and EVs over 18 h. Data are the means of three biological replicates ± SEM. (**D**) Western blotting analysis of EV protein OmpA after incubation for (2, 4, 6, 8, 10, 12, 14, 16, and 18 h). A representative western blot is shown. (**E**) SDS-PAGE analysis of proteinase-K-treated EVs, untreated EVs, and CT265 whole-cell bacterial lysate (WC). (**F**) Protein concentrations of proteinase-K-treated EVs and -untreated EVs. Statistical test: *t-*test; ^*^P < 0.05, ^**^P < 0.01. Data are the means of five biological replicates ± SEM.

To characterize APEC EVs, the EVs produced by APEC strain CT265 were isolated from the culture supernatant and purified with OptiPrep™ gradient ultracentrifugation. The morphology and size distribution of the EVs were determined with TEM and NTA. TEM showed that the EVs produced by APEC CT265 were spherical (Fig. 1A). NTA showed that the EVs produced by CT265 were heterogeneous in size, ranging from 20 to 200 nm, and the median/mean diameter of the EVs were approximately 117.4 nm (Fig. 1B). The concentration of the total EVs was 1.04 × 10^12^ particles/mL. To determine the kinetics of EV production associated with APEC strain CT265, bacteria were cultured in 1.0 L of LB broth, and 50 mL aliquots were collected every two hours. The EV pellets were isolated and purified with ultracentrifugation. To examine EV production, immunoblotting was used with an anti-OmpA antibody to analyze the EV signals, followed by densitometric quantification (DU). We simultaneously plotted a growth curve for APEC strain CT265, expressed in OD_600_. The intensity of the OmpA signals exactly paralleled the growth of strain CT265 from the logarithmic growth phase to the plateau phase (Fig. 1C and D). This indicated that the production of APEC EVs correlated positively with the growth of the bacterial culture, and that APEC released EVs continuously during its growth.

Typically, APEC EVs contain diverse molecular components, including proteins, DNA, and RNA, which play vital roles in information exchange and regulation among bacteria [37]. To examine the EV-associated proteins, purified proteinase-K-treated or -untreated EVs were analyzed with SDS-PAGE. The results showed that the EVs contained large amounts of protein. Protein was even present in the proteinase-K-treated EVs, although the content was lower (Fig. 1E F). As we know, the EV membrane protects the intramembrane proteins from degradation by proteinase K. Therefore, these results indicated that a large fraction of the EVs is made up of proteins which may be surface-bound or may be internal.

### TLR4 plays a vital role in macrophage immune response induced by EVs

**Fig. 2:**
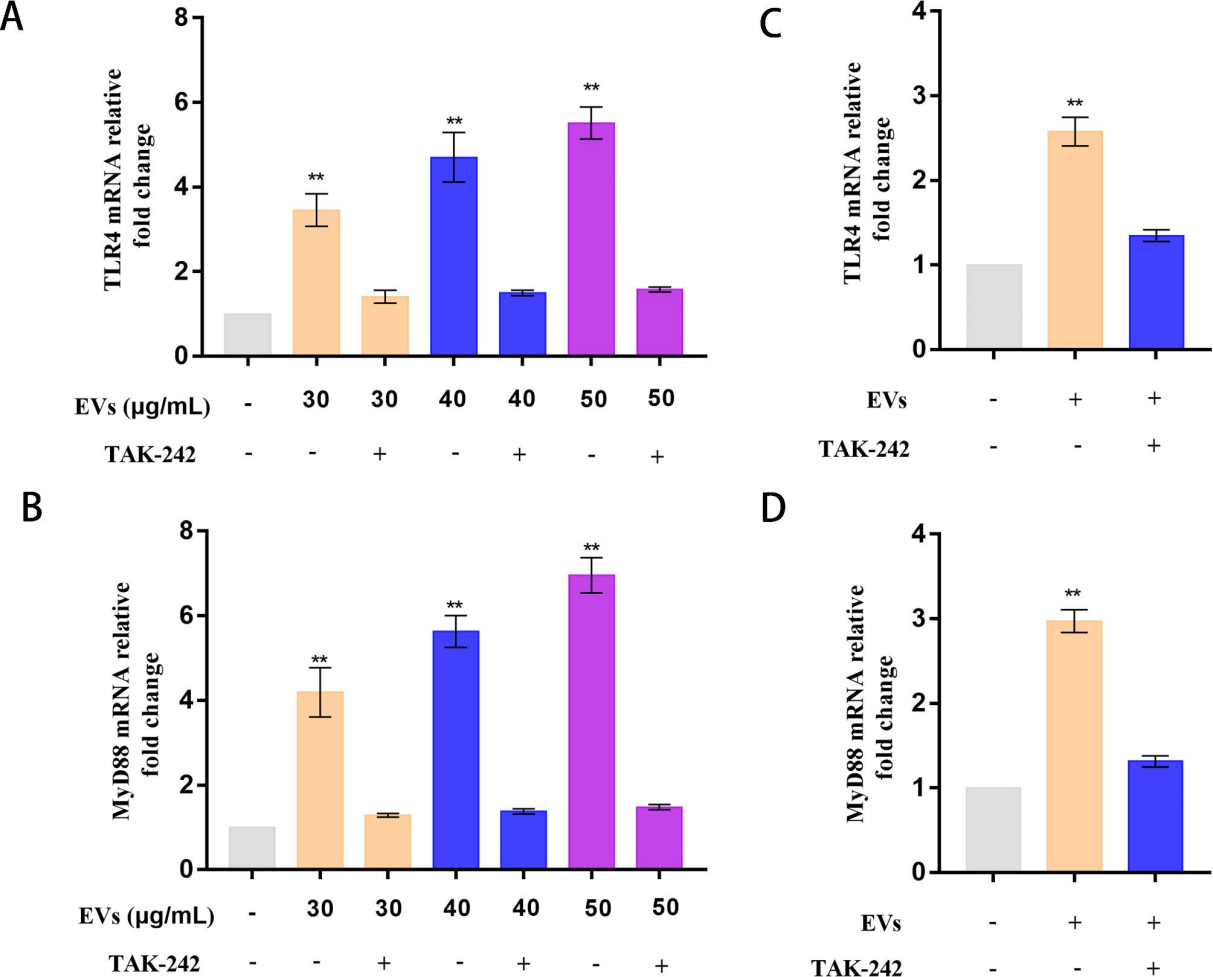
TLR4/MYD88 signaling pathway is involved in EV-induced inflammation in macrophages. (**A + B**) Levels of TLR4 and MYD88 mRNAs in PMA-treated THP-1 macrophages, pretreated or not pretreated with TAK242, and incubated for 12 h with EVs (30, 40, or 50 µg/mL) were measured with RT–qPCR. Statistical test: one-way ANOVA, ^*^P < 0.05, ^**^P < 0.01. (**C + D**) Levels of TLR4 and MYD88 mRNAs in HD11 macrophages incubated for 12 h with EVs (30 µg/mL) pretreated or not pretreated with TAK242 were measured with RT-qPCR. Statistical test: one-way ANOVA, ^*^P < 0.05, ^**^P < 0.01

As on the bacterial membrane, many biomolecules occur on the membrane surface of EVs, including the outer-membrane proteins and LPS, which play vital roles in the interaction of EVs with their host immune cells [38]. Acting as a pathogen-associated molecular pattern (PAMP), LPS binds to TLR4 on the surfaces of macrophages to activate the host immune responses. In this study, we investigated whether TLR4 affects the cell immune responses by interacting with APEC EVs. To investigate this, PMA-differentiated THP-1 cells were treated or not treated with 20 µM TAK-242 (a TLR4 inhibitor) for 4 h and then incubated with EVs (30, 40, or 50 µg/mL) for 12 h. The mRNA levels of TLR4 and MYD88 in PMA-differentiated THP-1 macrophages after APEC EV treatment were significantly higher than in the naive PMA-differentiated THP-1 macrophages (*P* < 0.01), when analyzed with RT–qPCR (Fig. 2A and B). However, the TLR4 and MYD88 mRNA levels in the EVs + TAK-242-treated THP-1 cells were clearly lower than those in the PMA-differentiated THP-1 macrophages treated with APEC EVs. There was no significant difference in the TLR4 and MYD88 mRNA levels in the EVs + TAK-242 cells and the naive PMA-differentiated cells (*P* > 0.05) (Fig. 2A and B).

Then we examined the effects of APEC EVs on HD11 cells. HD11 cells were treated or not treated with 20 µM TAK-242 for 4 h and incubated with EVs (30 µg/mL) for 12 h. qPCR showed that the levels of TLR4 and MYD88 mRNAs increased after EV treatment, whereas there was a significant reduction in TLR4 and MYD88 mRNA after pretreatment with TAK-242 (Fig. 2C and D). These results demonstrated that APEC EVs activated the TLR4 signaling pathway, and that TAK-242 inhibited this process.

### TLR4/MYD88/NF-κB signaling pathway participates in the activation of the APEC-EV-induced NLRP3 inflammasome in THP-1 macrophages

**Fig. 3:**
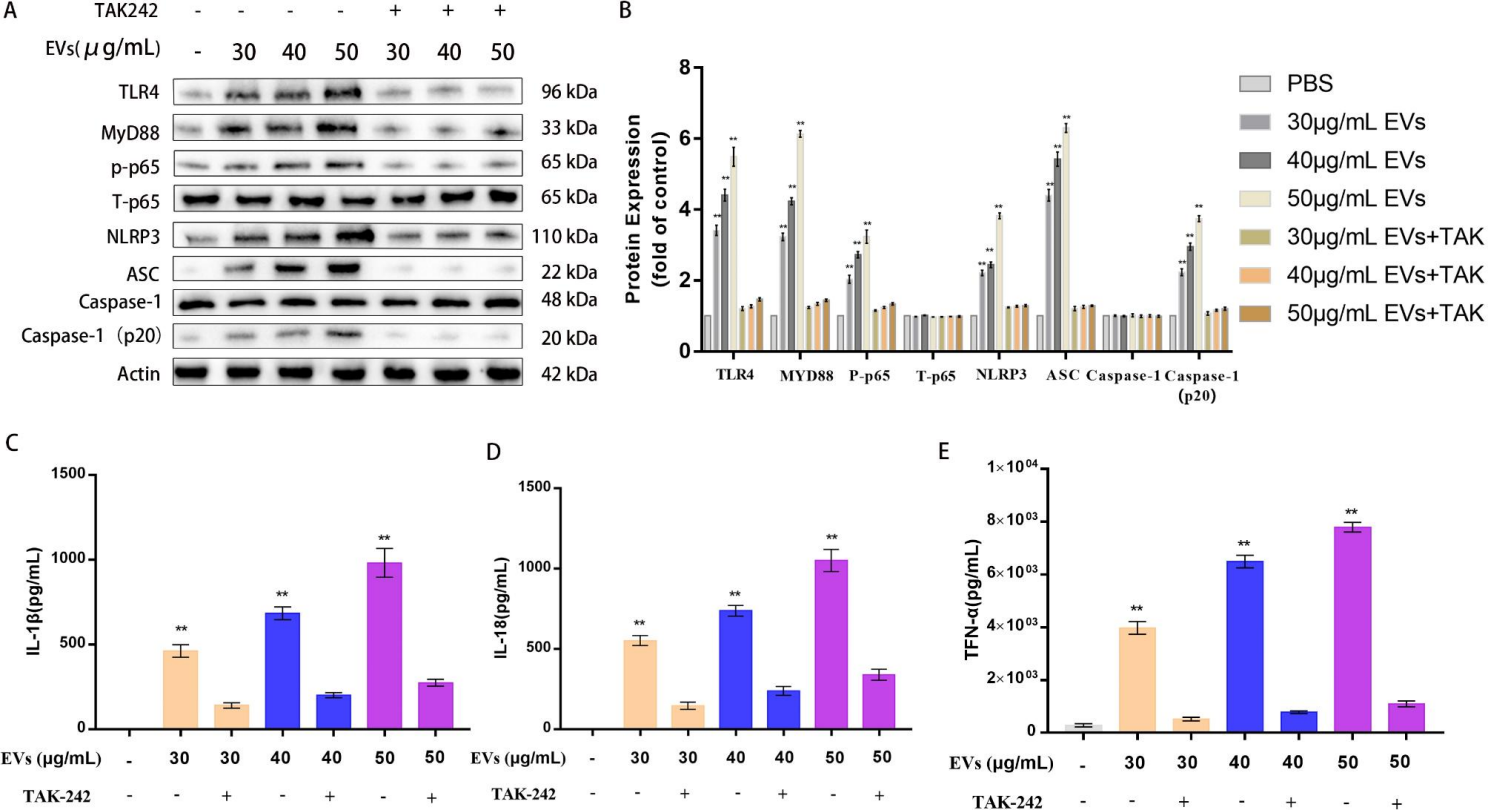
EVs induced an inflammatory response in macrophages. (**A**) Expression levels of inflammasome-related proteins in PMA-treated THP-1 macrophages, pretreated or not pretreated with TAK242 and incubated for 12 h with EVs (30, 40, or 50 µg/mL), were measured with a western blotting assay. A representative western blot is shown. (**B**) Densitometric analysis was used to quantify the levels of inflammasome-related proteins. Statistical test: two-way ANOVA, ^*^P < 0.05, ^**^P < 0.01. (**C + D + E**) PMA-treated THP-1 macrophages, pretreated or not pretreated with TAK242, were incubated for 12 h with EVs (30, 40, or 50 µg/mL), and the expression levels of IL1β, IL18, and TNF-α were determined with ELISAs. Statistical test: one-way ANOVA, ^*^P < 0.05, ^**^P < 0.01

A recent report has shown that both the canonical and noncanonicl NF-κB signaling pathways were involved in the host immune response [39]. Therefore, APEC EVs may induce the macrophage inflammatory response via an NF-κB-mediated immune response. Western blotting showed that the expression of the TLR4, MYD88, and NF-κB P-p65 proteins was significantly higher in APEC-EV-induced PMA-pretreated THP-1 macrophages than in the naive PMA-differentiated THP-1 cells (Fig. 3A and B).

The levels of proinflammatory cytokines were assessed with ELISAs. The levels of IL1β, IL18, and TNF-α in APEC-EV-induced THP-1 macrophages were significantly higher than in the naive PMA-differentiated macrophages (*P* < 0.01), whereas the levels of IL1β, IL18, and TNF-α in the EVs + TAK-242-treated THP-1 macrophages were clearly lower than in the EV-treated THP-1 macrophages (*P* < 0.01) (Fig. 3C and D, and 3E).

To determine whether the increased secretion of proinflammatory cytokines (IL1β and IL18) in THP-1 macrophages under APEC EV stimulation activates the NLRP3 inflammasome, we investigated the effect of EV treatment on the NLRP3 inflammasome. The NLRP3 inflammasome in the EV-treated THP-1 macrophages was clearly activated, evident as markedly increased levels of NLRP3, ASC, and caspase 1 (all *P* < 0.01) (Fig. 3A and B). The expression of NLRP3 is the rate-limiting step in the inflammasome activation process [40].

Compared with protein amounts, the expression of the TLR4, MYD88, and NF-κB P-p65 proteins in the EVs + TAK-242-treated THP-1 macrophages was markedly lower than in the APEC-EV-treated THP-1 macrophages, and the expression of NLRP3, ASC, and caspase 1, which reflects the activation of the NLRP3 inflammasome, was also significantly reduced (Fig. 3A and B). There were no obvious differences in the expression of these proteins between the EVs + TAK-242-treated THP-1 macrophages and the naive PMA-differentiated macrophages (Fig. 3A and B). These results suggested that the NLRP3 inflammasome was activated by APEC EVs via the TLR4/MYD88/NF-κB signaling pathway in THP-1 macrophages.

### LPS loss from APEC EVs reduced the activation of the NLRP3 inflammasome via TLR4/MYD88/NF-κB signaling

**Fig. 4:**
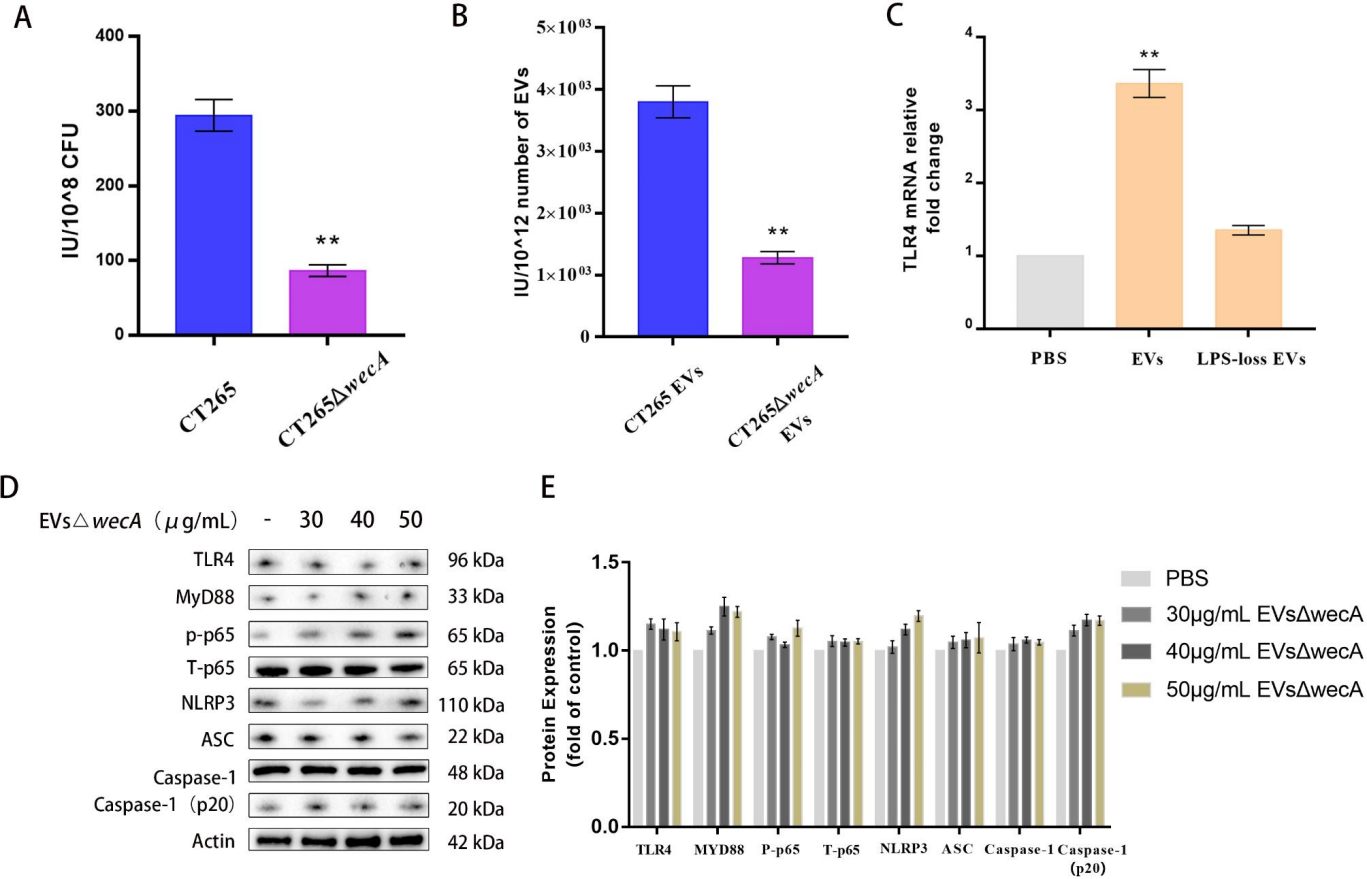
Loss of LPS significantly weakened the inflammatory response in THP-1 macrophages. (**A + B**) *Escherichia coli* CT265, mutant strain CT265Δ*wecA*, and EVs from both bacteria were analyzed with the PyroGene™ Recombinant Factor C Endotoxin Detection Assay. Statistical test: one-way ANOVA, ^*^P < 0.05, ^**^P < 0.01. (**C**) TLR4 mRNA levels in PMA-treated THP-1 macrophages, pretreated or not pretreated with TAK242 and incubated for 12 h with EVs lacking LPS (30 µg/mL), were measured with RT–qPCR. Statistical test: one-way ANOVA, ^*^P < 0.05, ^**^P < 0.01. (**D**) Expression levels of inflammasome-related proteins in PMA-treated THP-1 macrophages incubated for 12 h with CT265*ΔwecA* EVs (30, 40, or 50 µg/mL) were measured with western blotting assays. A representative western blot is shown. (**E**) Densitometric analysis was used to quantify the levels of inflammasome-related proteins. Statistical test: two-way ANOVA, ^*^P < 0.05, ^**^P < 0.01

To determine whether the LPS on APEC EVs activates the NLRP3 inflammasome in THP-1 macrophages, we removed the LPS from APEC EVs as follows. The *wecA* gene encodes the O-antigen transferase that initiates the attachment of the *E. coli* O-antigen to polysaccharides, and the deletion of the *wecA* gene causes the loss of *E. coli* LPS [41, 42]. Therefore, we truncated the LPS of APEC strain CT265 by deleting the *wecA* gene and thus removing the O-antigen. In this study, the *wecA* gene was deleted from APEC strain CT265 by homologous recombination. We tested the production of LPS in wild-type strain CT265 and mutant strain CT265Δ*wecA*. As shown in Fig. 4A, the amount of LPS extracted from the same number of bacteria (1.0 × 10^8^ CFU) of these two strains differed markedly, and the production of LPS in the mutant strain CT265Δ*wecA* was significantly lower than that in wild-type strain CT265. We also extracted the LPS from the same number of EVs (~ 1.0 × 10^12^) produced by wild-type CT265 and mutant CT265Δ*wecA*. Significantly less LPS was extracted from the EVs of mutant CT265Δ*wecA* was than from those of wild-type strain CT265 (Fig. 4B).

We then compared the effect of EVs with or without LPS on the activation of the proinflammatory response of macrophages. RT–qPCR showed that the amount of TLR4 mRNA in PMA-differentiated THP-1 macrophages treated with EVs (30 µg/mL) lacking LPS was significantly lower than that in macrophages treated with normal APEC EVs but close to that in the naive PMA-differentiated THP-1 macrophages (*P* < 0.01) (Fig. 4C). Similarly, a western blotting analysis indicated that the expression of proteins TLR4, MYD88, and NF-κB P-p65 was significantly reduced in THP-1 macrophages treated with EVs lacking LPS, but was close to that in the naive PMA-differentiated THP-1 macrophages (Fig. 4D and E). Furthermore, the NLRP3 inflammasome in the THP-1 macrophages treated with EVs lacking LPS was not obviously activated, evident as the reduced levels of NLRP3, ASC, and caspase 1 detected (Fig. 4D and E). The expression of TLR4, MYD88, and NF-κB P-p65 proteins in THP-1 macrophages treated with EVs lacking LPS was no changes than in the naive EV-treated THP-1 macrophages, and the expression of NLRP3, ASC, and caspase 1 was also no changes (Fig. 4D and E), indicating that APEC EVs promote the activation of the NLRP3 inflammasome via LPS.

### APEC EVs induce NETs in murine peripheral blood neutrophils

**Fig. 5:**
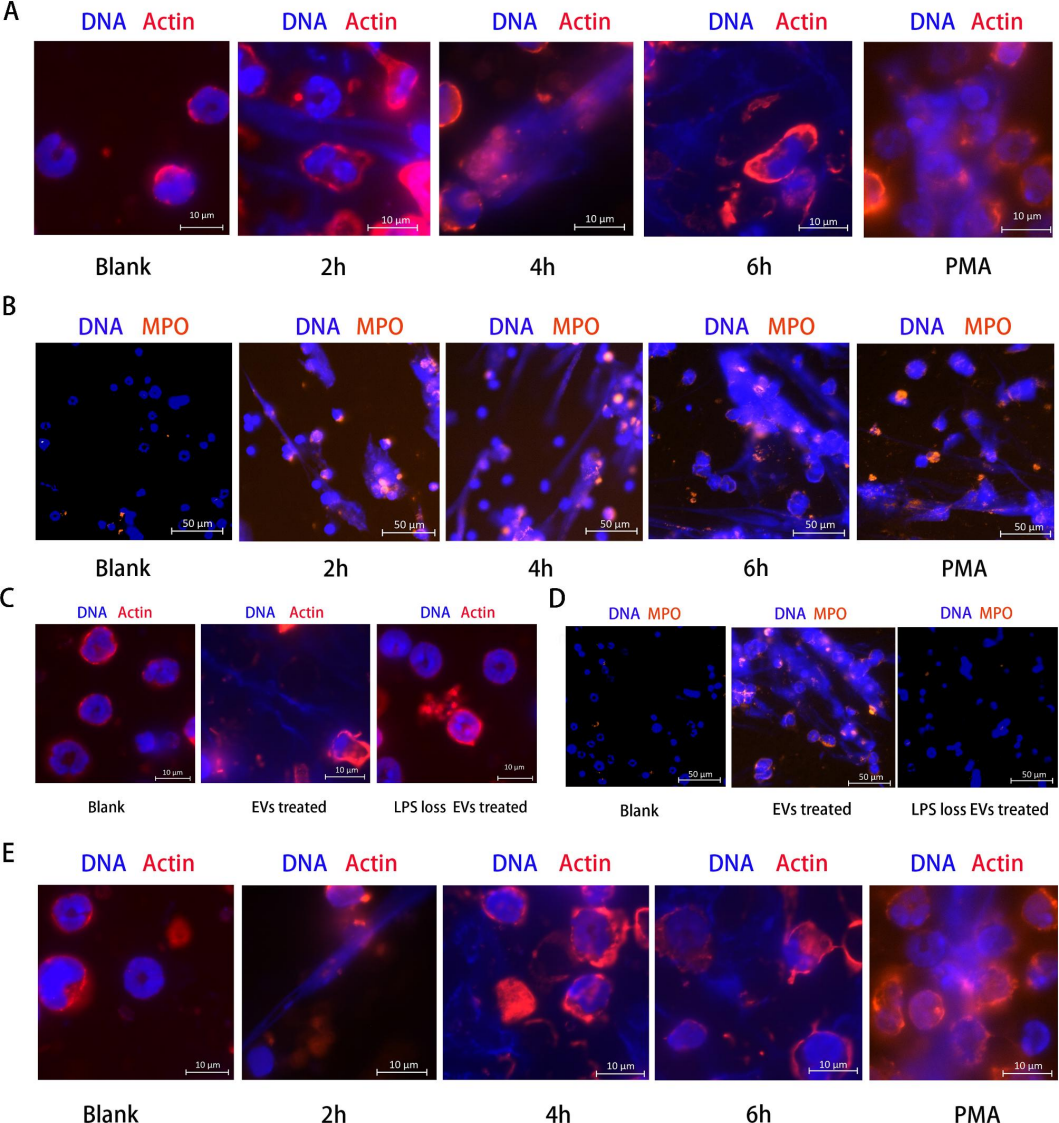
Neutrophil activation by EVs led to the formation of neutrophil extracellular traps (NETs). (**A**) Formation of NETs in mouse bone-marrow neutrophils activated with 50 µg/mL EVs for different times (2, 4, or 6 h). Cells were visualized with fluorescence microscopy. 100nM PMA as the positive control. (**B**) Detection of myeloperoxidase (MPO) protein in mouse bone-marrow neutrophils incubated with 50 µg/mL EVs. (**C**) After incubation with 50 µg/mL EVs lacking LPS for 6 h, neutrophils were visualized with fluorescence microscopy. No NETs were observed. (**D**) Neutrophils were incubated with 50 µg/mL EVs lacking LPS for 6 h, and visualized with fluorescence microscopy. No MPO protein was detected in neutrophils treated with EVs lacking LPS. (**E**) Formation of HETs in chicken heterophils after activation with 50 µg/mL EVs for different times (2, 4, or 6 h). Data represent one of three independent experiments. Cells were fixed and stained with DAPI (blue) and MPO antibody (orange). Data represent one of three independent experiments

In this study, we demonstrated that the EVs produced by APEC activated the macrophage inflammatory response and their release of cytokines and chemokines. We speculated that during extraintestinal APEC infection, the interaction between APEC EVs and macrophages may activate and promote neutrophil migration. To examine the interaction between APEC EVs and murine neutrophils, EVs (50 µg/mL) were incubated with neutrophils isolated from murine bone marrow. At three time points (after incubation for 2, 4, or 6 h), the EV-treated neutrophils were permeabilized, fixed, and incubated with antibodies. NET formation was observed with fluorescent imaging. The NET DNA backbone around the neutrophils was observed in both APEC-EV-treated and PMA-stimulated neutrophils (Fig. 5A), and EVs induced NETs in neutrophils in a time-dependent manner (Fig. 5A). After treatment with EVs, murine peripheral blood neutrophils were induced to release NETs at 2 h, indicating that peripheral blood murine neutrophils are readily activated by external stimuli. APEC-EV-induced NET formation was not a rapid reaction (within 15 min), suggesting that the APEC-EV-induced release of NETs might belong to the classic within 2 to 4 h model. To further investigate the NET formation induced by APEC EVs, we measured the levels of MPO protein, a recognized biomarker of NETs. Immunofluorescent signals for MPO protein were detected in APEC-EV-treated neutrophils (Fig. 5B), demonstrating that EVs stimulate neutrophils to generate NETs, as does PMA (Fig. 5B).

We then compared the effect of EVs containing or lacking LPS on the activation of NETs in neutrophils. No NET DNA backbone was observed around neutrophils treated with EVs lacking LPS (Fig. 5C), and no immunofluorescent signals for MPO were detected in those neutrophils (Fig. 5D). These results indicated that APEC EVs promoted the activation of NETs via LPS.

### APEC EVs activate HET formation in chicken peripheral blood heterophils

To investigate the interaction between APEC EVs and heterophils, EVs (50 µg/mL) were incubated with chicken heterophils. After treatment with EVs for various times (2, 4, or 6 h), the heterophils were permeabilized, fixed, and observed with fluorescence microscopy. The APEC EVs induced the release of HETs from heterophils in a time-dependent manner (Fig. 5E). The chicken heterophils produced HETs at 2 h after treatment with EVs, indicating that heterophils were also readily activated by external stimuli to release HETs.

### APEC EV treatment induces SAPK/JNK activation in neutrophils

**Fig. 6:**
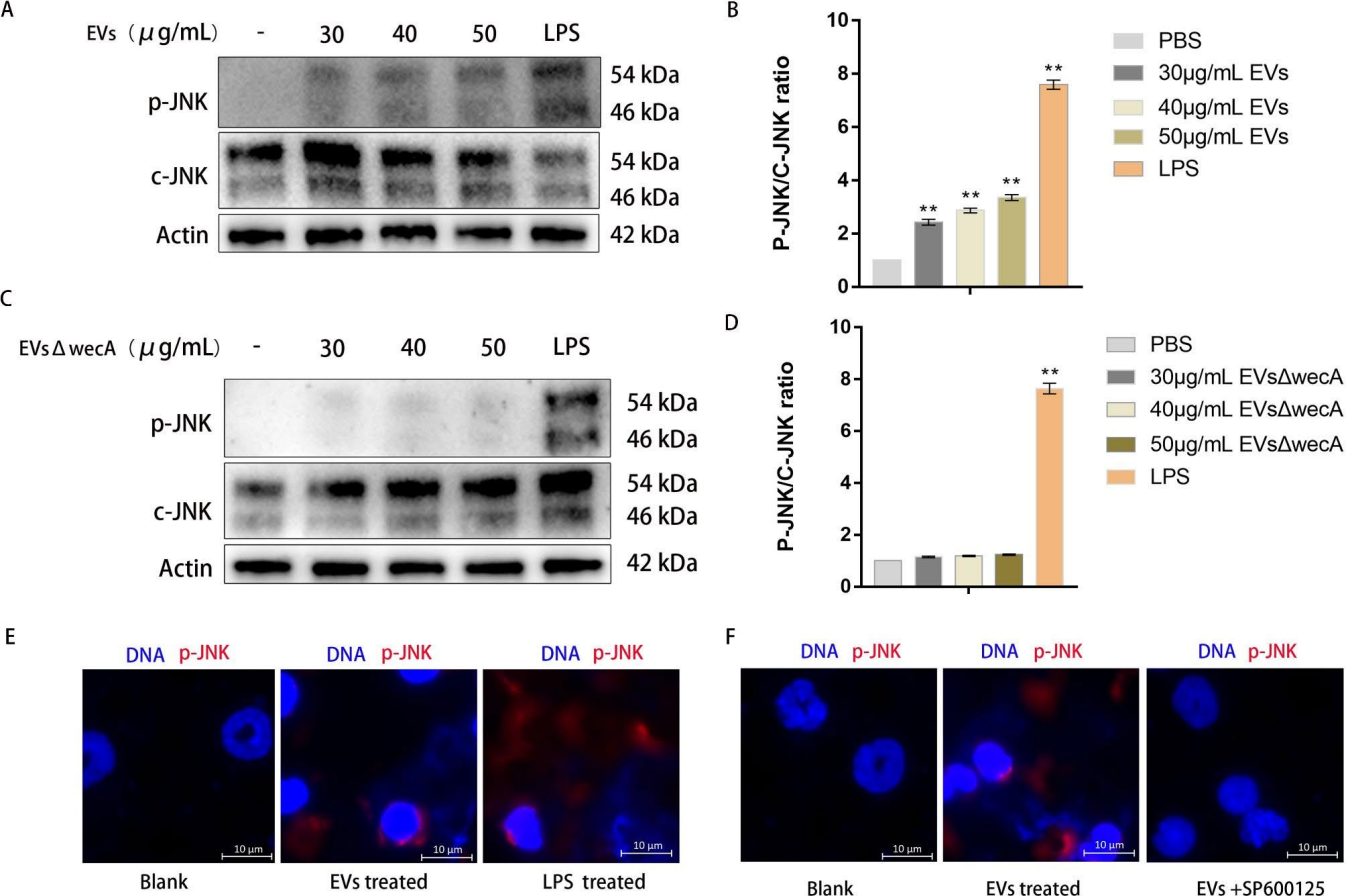
JNK inhibition suppressed the formation of APEC-EV-mediated NETs. (**A**) Levels ofp-JNK1 and p-JNK2 in mouse neutrophils treated with EVs (30, 40, or 50 µg/mL) for 30 min were measured with western blotting assays. A representative western blot is shown. 50 µg/mL of LPS as the positive control. (**B**) Densitometric analysis was used to quantify the protein levels of p-JNK1 and p-JNK2. Statistical test: one-way ANOVA, ^*^P < 0.05, ^**^P < 0.01. (**C**) Levels of p-JNK1 and p-JNK2 in mouse neutrophils treated with EVs (30, 40, or 50 µg/mL) for different times 30 min were measured with western blotting assays. A representative western blot is shown. (**D**) Densitometric analysis was used to quantify the protein levels of p-JNK1 and p-JNK2. Statistical test: one-way ANOVA, ^*^P < 0.05, ^**^P < 0.01. (**E + F**) APEC CT265 EVs (50 µg/mL) were incubated with mouse neutrophils for 120 min. Fluorescence microscopy showed that neutrophils released NETs with p-JNK, but the SAPK/JNK inhibitor SP600125 inhibited the EV-mediated formation of NETs.

To determine the relevance of SAPK/JNK in APEC-EV-induced NETosis, we examined the effect of APEC EVs on SAPK/JNK activation in mouse neutrophils. As shown in Fig. 6A, western blotting analyses showed that in neutrophils incubated with different concentrations of APEC EVs (30, 40, or 50 µg/mL) for 30 min, SAPK/JNK was phosphorylated (p-SAPK/JNK) to different levels. The phosphorylation of both SAPK/JNK-1 and SAPK/JNK-2 was detected as the concentration of APEC EVs increased (Fig. 6A and B). After treatment with 30 µg/mL APEC EVs, the phosphorylation of SAPK/JNK was relatively low, whereas after treatment with 40 or 50 µg/mL EVs, SAPK/JNK activation was consistently higher. However, the phosphorylation of SAPK/JNK was not detected in neutrophils treated with EVs lacking LPS (Fig. 6C and D), suggesting that the LPS contained in the APEC EVs induces the activation of SAPK/JNK in neutrophils. The activation of SAPK/JNK in neutrophils treated with APEC EVs was detected on confocal microscopic images after immunostaining for p-SAPK/JNK (red) (Fig. 6E). The results showed that neutrophil SAPK/JNK was activated by APEC EVs in a dose-dependent manner. To determine the role of SAPK/JNK in NET formation, we examined the NET release in the presence or absence of the SAPK/JNK inhibitor SP600125 in confocal microscopic images. No NET release was induced by APEC EVs in the presence of SP600125(Fig. 6F). These results demonstrated that APEC-EV-mediated NET formation was dose-dependently suppressed by an SAPK/JNK inhibitor.

### Inhibition of TLR4 signaling suppresses APEC-EV-induced NET formation

**Fig. 7:**
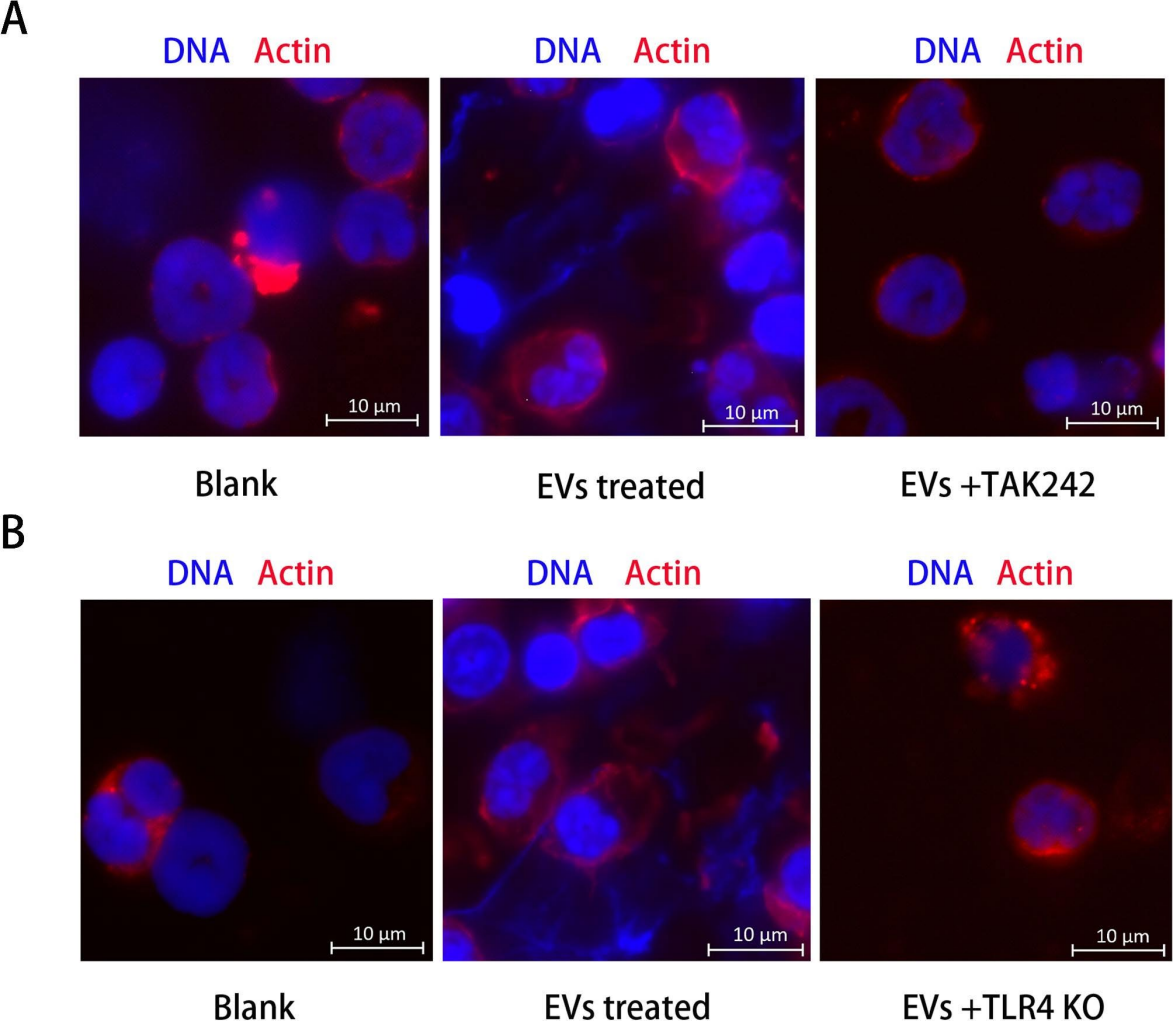
Inhibition of TLR4 suppressed NET formation in neutrophils. (**A**) CT265 EVs (50 µg/mL) were incubated with TAK242-pretreated mouse neutrophils for 120 min. Fluorescence microscopy showed that the neutrophils did not release NETs. (**B**) CT265 EVs (50 µg/mL) were incubated with TLR4 knockout (KO) mice neutrophils for 120 min. Fluorescence microscopy showed that the neutrophils did not release NETs. Neutrophils from TLR4 KO mice did not support NET formation

To determine the role of TLR4 signaling in APEC-EV-induced NET formation, neutrophils were pretreated with different concentrations of the TLR4 inhibitor TAK242, and then incubated with APEC EVs (50 µg/mL) or LPS **(**50 µg/mL**)** to induce NET release. Immunofluorescent images showed that the TLR4 inhibitor suppressed the APEC-EV-mediated formation of NETs (Fig. 7A), indicating that TLR4 signaling was important in APEC-EV-mediated NET formation. To further investigate the function of TLR4 signaling in APEC-EV-induced NETs formation, the neutrophils were isolated from TLR4 knockout (KO) mice and treated with APEC EVs. As shown in Fig. 7B, when stimulated with APEC EVs, neutrophils from TLR4 KO mice were less likely to release NETs and stimulate the phosphorylation of JNK/SAPK than those from control mice. Therefore, APEC EVs were recognized by TLR4, which subsequently activated JNK/SAPK to promote NET release.

### APEC EVs cause macrophage damage in a dose- and time-dependent manner

**Fig. 8:**
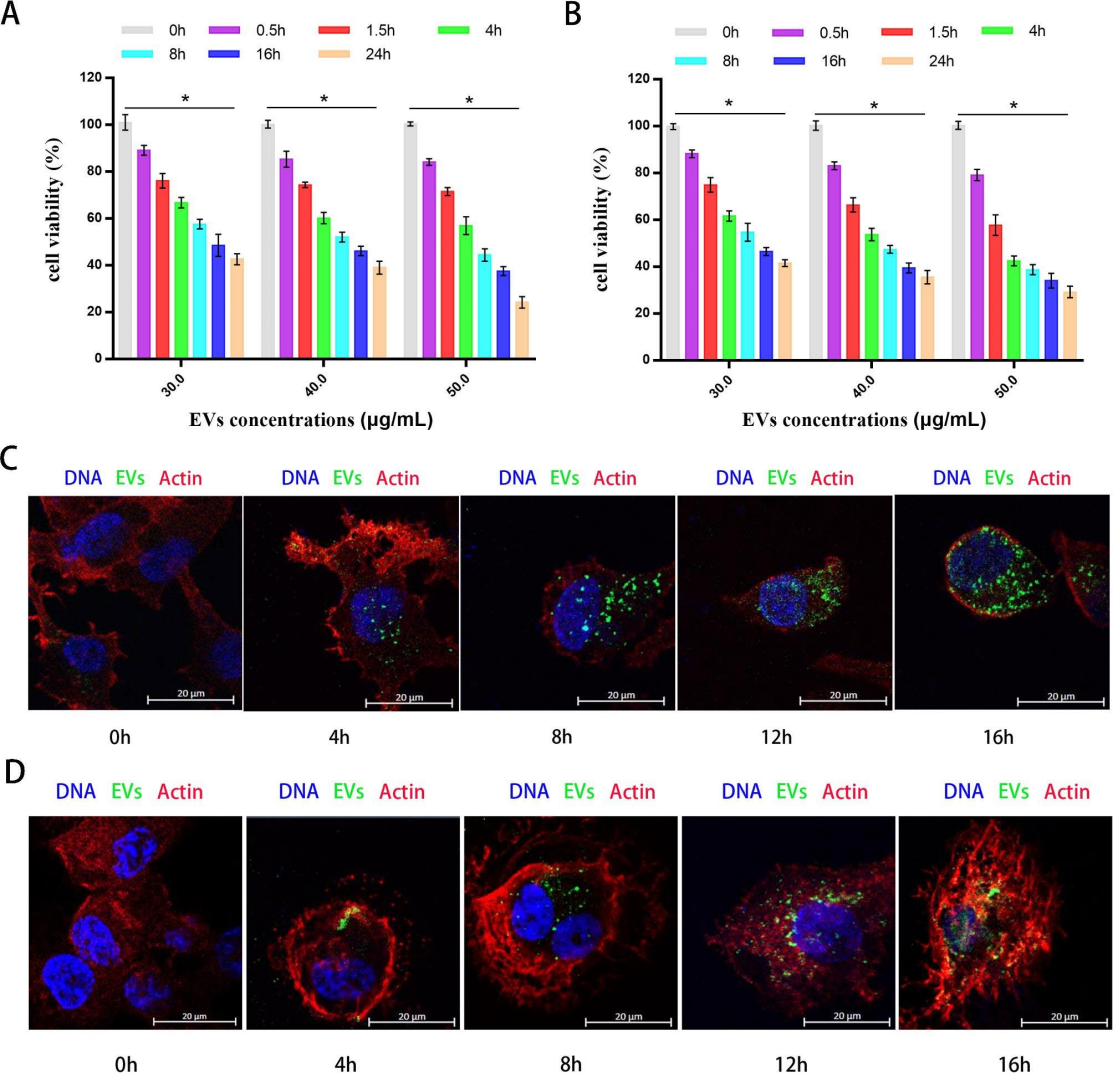
CT265 EVs are cytotoxic to macrophages and are taken up by macrophages. (**A** and **B**) HD11 and PMA-activated THP-1 macrophages were incubated with different concentrations of CT265 EVs (30, 40, or 50 µg/mL) for 24 h. Cell viability was determined with a CCK-8 assay. At each time point, data were normalized to the untreated control. Data are shown as the means ± SEM of three independent experiments. Statistics: one-way ANOVA, ^*^P < 0.05, ^**^P < 0.01 compared with no treatment. Data are the means of three biological replicates ± SEM. (C and D) HD11 and PMA-activated THP-1 macrophages were treated with 50 µg/mL DiO-labeled EVs and incubated for 4, 8, 12, or 16 h. The cells were fixed and stained with DAPI (blue) and phalloidin (red). The figure represents one of three independent experiments

Macrophages are very important immune cells and constitute an important line of defense in the body’s immune response [43]. To establish chronic infections in the body, pathogens must evade capture by macrophages. In a previous study, we showed that APEC replicates and proliferates in chicken macrophage HD11 cells [11], and several studies have shown that *E. coli*-produced EVs cause cell injury [44]. Therefore, having established that APEC EVs activated the immune response of macrophages and initiate NET formation, we also investigated whether they damage the immune function of macrophages. To do so, different concentrations of CT265 EVs (30, 40, or 50 µg/mL) were incubated with HD11 or THP-1 macrophages for 24 h, and then the cytotoxic effect of the EVs on the macrophages was measured with a CCK-8 kit at various times (0, 0.5, 1.5, 4, 8, 16, or 24 h). The APEC EVs showed excellent concentration- and time-dependent cytotoxicity against both types of macrophages (Fig. 8A and B), suggesting that EVs from APEC CT265 carried specific virulence factors that caused cytotoxicity.

To further investigate the interaction between CT265 EVs and macrophages and to determine whether EVs are internalized by macrophages, purified DiO-labeled EVs (50 µg/mL) were incubated with THP-1 or HD11 macrophages on confocal dishes. After incubation for various times (4, 8, 12, or 16 h), macrophages containing internalized APEC EVs were observed with laser confocal microscopy. As shown in Fig. 8C and D, the numbers of EVs internalized by the HD11 and THP-1 cells increased over time, up to 12 and 16 h, respectively, when the internalization of EVs by the macrophages had reached a plateau. Overall, this study demonstrated that APEC EVs were taken-up by different types of macrophages.

### APEC EVs contribute to macrophages death

**Fig. 9:**
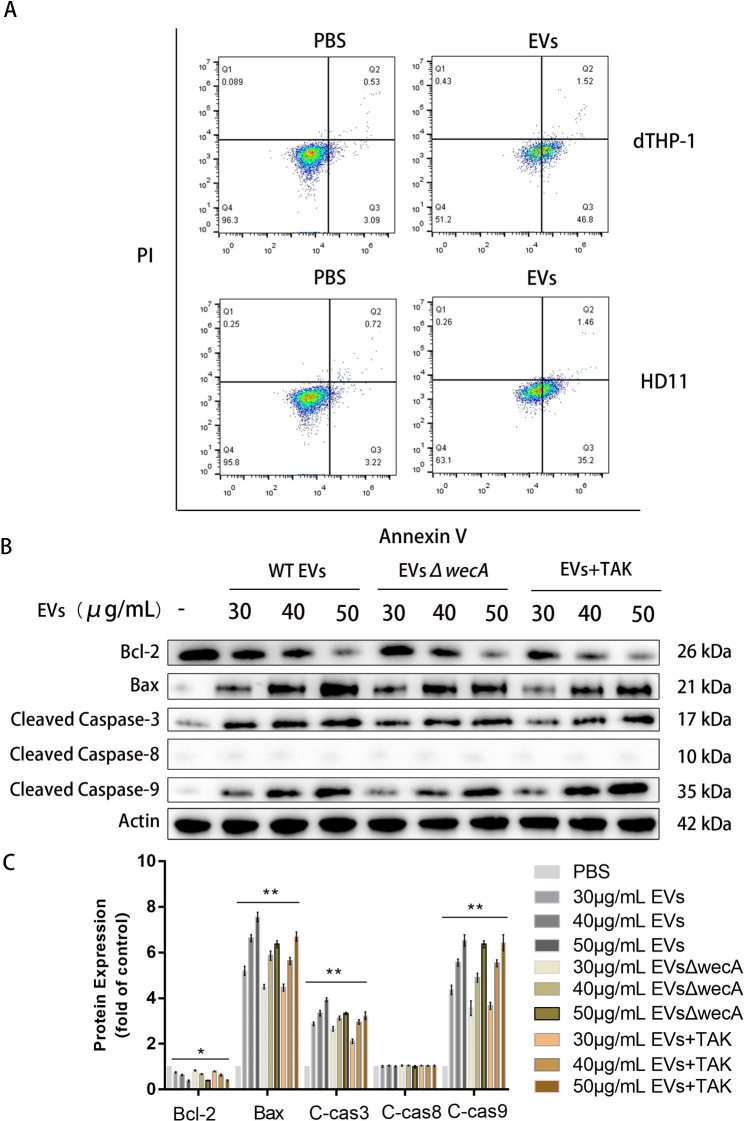
Apoptosis of THP-1 macrophages treated with APEC EVs. (**A**) PMA-activated THP-1 and HD11 cells were incubated with CT265 EVs (50 µg/mL) for 12 h, and apoptosis was detected with annexin V–FITC flow cytometry. The results shown are representative of one experiment with three replicates. (**B**) EVs (30, 40, or 50 µg/mL) from APEC CT265 or CT265*ΔwecA* were incubated with PMA-activated THP-1 macrophages for 12 h. EVs (30, 40, or 50 µg/mL) from CT265 were incubated with TAK242-pretreated PMA-activated THP-1 macrophages for 12 h. The cells were collected and the expression of apoptosis-related proteins detected with western blotting. The results shown are representative of one experiment with three replicates. (**C**) Densitometric analysis was used to quantify the levels of apoptosis-related proteins. Statistical test: two-way ANOVA, ^*^P < 0.05, ^**^P < 0.01

Previous studies have shown that EVs from Gram-negative bacteria caused apoptosis in bone-marrow derived macrophages [45]. Because APEC EVs induced damage in THP-1 and HD11 macrophages, we wondered whether EVs induced apoptosis in macrophages. To investigate this, macrophages were incubated with 50 µg/mL APEC EVs for 12 h, the cells were collected, and cell death was detected with flow cytometry. As shown in Fig. 9A, EV-treated THP-1 and HD11 cells showed significantly more apoptosis than cells not treated with EVs.

To further investigate the effect of EVs on macrophage apoptosis, we measured the levels of apoptosis-related proteins, cleaved caspase 3, cleaved caspase 8, cleaved caspase 9, BAX, and BCL2 in THP-1 macrophages. Western blotting showed elevated levels of the apoptosis-related proteins cleaved caspase 3 and caspase 9 and proapoptotic protein BAX, and reduced levels of antiapoptotic protein BCL2 (Fig. 9B C). These results are consistent with the flow-cytometric results, confirming that APEC EVs cause apoptosis in macrophages. However, the levels of cleaved caspase 8 were not significantly altered by treatment with EVs. Cleaved caspase 8 is one of the key molecules in the extrinsic apoptosis pathway, so we speculated that APEC EVs initiate apoptosis via the intrinsic pathway, rather than via the extrinsic pathway, in THP-1 cells.

To evaluate the effects of LPS on apoptosis, THP-1 macrophages were incubated with 50 µg/mL EVs produced by wild-type APEC CT265 or mutant APEC CT265Δ*wecA*. After incubation for 12 h, the macrophages were collected, and western blotting performed to detect protein expression. The loss of LPS on the surfaces of the EVs did not significantly reduce macrophage apoptosis (Fig. 9B C). Because TLR4 is the canonical membrane receptor for LPS, we next examined whether TLR4 was involved in EV-induced apoptosis. Macrophages pretreated with TAK-242 were incubated with EVs from wild-type APEC CT265 for 12 h, and then subjected to a western blotting analysis. As shown in (Fig. 9B C) there was no significant difference in the expression levels of apoptosis-related proteins between the TAK-242-treated and -untreated groups, indicating that neither LPS nor TLR4 was critical for APEC-EV-induced macrophage apoptosis. Previous studies have shown that EV-associated virulence proteins, such as enterohemorrhagic *E. coli* (EHEC) Hly and Stx2a, induce apoptosis in epithelial and endothelial cells [46, 47]. Therefore, we speculated that the macrophage damage caused by APEC EVs was predominantly caused by virulence factors carried within the EVs. Having demonstrated that the loss of LPS from APEC EVs and the inhibition of TLR4 reduced the proinflammatory responses of macrophages, we concluded that APEC EVs cause apoptosis in macrophages, and that the loss of LPS from APEC EVs did not prevent this process, whereas it reduced the proinflammatory response of macrophages and NET formation by neutrophils.

## Discussion

In recent years, with the increasing prevalence of antimicrobial resistance and increasing virulence, APEC, a foodborne pathogen, has become a serious worldwide public health concern [48, 49]. To better prevent and control APEC infections, the pathogenic mechanisms of APEC must be clarified. The numerous studies of APEC pathogenicity have mainly focused on the virulence factors located on the bacterial membrane surface and the virulence-related transcriptional regulators carried by the bacteria [50–52]. The EVs released by bacteria carry many molecular cargoes, including DNA, RNA, LPS, cytoplasmic proteins, and outer membrane proteins [53, 54]. The cargoes are encapsulated within the EV, and then transmitted to host cells during the interaction between EVs and the host cells. Like the naive parent bacteria, EVs have a lipid bilayer membrane structure to protect their cargo from degradation and destruction by external factors. Like many other bacteria, APEC releases EVs to the external environment [55].

Previous studies have shown that APEC was a successful pathogen with zoonotic potential [4–6]. Therefore, we wondered whether APEC EVs affect or disturb the host’s immune responses. We have demonstrated that APEC EVs were sensed by the host’s innate immune response, and induced both HD11 and THP-1 inflammatory responses. A previous study indicated that EVs derived from different bacterial sources activated the production of cytokines in host cells [22]. The innate immune response is an important part of the body’s immune system, playing a significant role in the host’s defenses against bacterial infection [56]. TLRs identify PAMPs and are crucial in the innate immune response [57]. Typically, TLRs occur on the surface of the macrophage membrane and act as a link between the innate and adaptive immunities [58]. Invading bacteria are first recognized by TLRs, which then activate the body’s inflammatory response. In this study, we demonstrated the role of TLR4 in the inflammatory response induced by APEC EVs. Our results showed that treatment with APEC EVs increased the TLR4 mRNA and protein levels in PMA-activated THP-1 macrophages, suggesting that TLR4 was involved in the immune response to APEC EVs. Pretreatment with TAK242 inhibited the TLR4-related inflammatory response activated by APEC EVs, indicating that when macrophages interacted with APEC EVs, the inflammatory response was dependent on the TLR4 signaling pathway. Our results also showed that APEC EVs enhanced the release of proinflammatory cytokines (IL6, IL8, and TNF-α) from THP-1 and HD11 macrophages, and increased the levels of NF-κB P-p65, MYD88, and NLRP3 in THP-1 cells. We speculated that APEC EVs activated the MYD88 and NF-κB signaling pathway to trigger the macrophage inflammatory response.

We have also shown that the inflammatory response in APEC-EV-treated THP-1 and HD11 macrophages involves the TLR4/MYD88/NF-κB signaling pathway, which triggers the NLRP3 inflammasome to stimulate a proinflammatory cascade and the release of proinflammatory factors [59, 60]. The TLR4 inhibitor TAK-242 markedly reduced TLR4 expression in macrophages after treatment with APEC EVs. Therefore, the effect of TAK-242 on the innate immune response in APEC-EV-treated macrophages was evaluated to assess the role of the TLR4/MYD88/NF-κB signaling pathway. Blocking TLR4/MYD88/NF-κB signaling reduced the release of proinflammatory IL1β, IL18, and TNF-α when macrophages interacted with APEC EVs. Our findings provided insight into the molecular mechanisms of the APEC-EV-induced inflammatory response in macrophages.

TLR4 is a critical receptor for LPS, which predominantly induces the inflammatory response through TLR4. TLR4/MYD88/NF-κB signaling is involved in LPS-induced inflammation via the activation of the NLRP3 inflammasome. The production mechanism of APEC EVs indicates that they carry bacterial LPS and cell membrane components [21]. The deletion of the *wecA* gene significantly reduced the LPS content in APEC and its EVs. When macrophages interacted with APEC EVs that contained no LPS, they did not produce a significant proinflammatory response and TLR4/MYD88/NF-κB signaling was not activated, indicating that the proinflammatory response of macrophages activated by APEC EVs was mediated by the LPS carried by the EVs, which bound to macrophage TLR4 and activated TLR4/MYD88/NF-κB signaling. Therefore, our study demonstrated that APEC EVs carrying LPS exerted a strong proinflammatory effect.

Neutrophils are activated through various cell-surface receptors, and the TLRs are essential pattern-recognition receptors (PRRs), which trigger the innate immune defenses by recognizing pathogens [61, 62]. The binding of LPS to membrane receptors TLR2 and TLR4 modulates several neutrophil immune responses, including NET formation [63]. NET formation is associated with the strong expression of TLR4 in neutrophils [64]. The formation mechanisms and compositions of NETs differ according to the stimulus received. TLR4 facilitates neutrophil responses to the LPS of Gram-negative bacteria [65], and TLR4 signaling is considered the critical trigger for the formation of LPS-induced NETs [66]. The activation of the signals required for NET formation is complex and diverse because several different stimuli are involved. It has been reported that PMA induced NET formation via RAF/MEK/ERK signaling [67]. The LPS in Gram-negative bacteria (*E. coli* and *Pseudomonas aeruginosa*) induced NET formation by activating SAPK/JNK signaling [66]. The LPS-induced activation of the JNK/SAPK signal to release NETs occurs downstream from the generation of reactive oxygen species (ROS) [68]. Blocking TLR4 signaling suppresses the activation of JNK/SAPK in the process of LPS-stimulated NET formation [66].

In this study, we have shown that TLR4 was involved in the NET formation induced in neutrophils by APEC EVs. Importantly, mouse bone-marrow neutrophils released NETs in a time-dependent manner after interaction with APEC-strain-CT265-derived EVs. APEC-EV-induced TLR4 signaling was demonstrated, and blocking TLR4 signaling suppressed APEC-EV-induced SAPK/JNK activation and NET release. We have also shown that JNK/SAPK was dose-dependently activated in neutrophils by APEC EVs. A JNK/SAPK inhibitor, SP600125, and a TLR4 inhibitor, TAK242, suppressed NET formation after APEC EV treatment. Therefore, we propose that NET formation in neutrophils in response to increasing concentrations of APEC EVs is initiated by a TLR4-dependent and SAPK/JNK-mediated signaling pathway. The inhibitor TAK242 blocks the interaction between TLR4 and adaptors TIRAP and TRAM, and LPS utilizes the TIRAP branch of the TLR4 signaling pathway to control NET formation [69]. Furthermore, TLR4/TIRAP signaling activates the MAPK cascade, including MAPK kinases (MKKs) 4 and 7, which subsequently stimulate SAPK/JNK [70]. LPS activates NET formation via TLR4/TIRAP/SAPK/JNK signaling. Therefore, we speculated that the TLR4/TIRAP/SAPK/JNK signaling pathway was responsible for APEC-EV-induced NET formation.

A previous study demonstrated that EVs derived from EHEC are internalized by human brain microvascular endothelial cells and Caco-2 cells [46]. It has also been reported that EVs derived from *L. pneumophila* were internalized by THP-1 macrophages [71]. After entering the host cells, EVs induce a strong immune response [22]. Our research has shown that APEC EVs are also internalized by macrophages (both HD11 and PMA-activated THP-1). A CCK-8 assay indicated that APEC EVs cause concentration- and time-dependent toxicity to macrophages (HD11 and PMA-activated THP-1). This is consistent with previous research that demonstrated that EVs reduce the viability of host cells [22]. We speculated that APEC EVs carried toxins, virulence proteins, or effectors to damage macrophages. Moreover, APEC EVs induced strong cytotoxicity for 24 h, which might be attributable to the different strains. ST117 strain CT265 is a virulent APEC strain and the EVs produced by ST117 isolates contain more virulence factors than the EVs of other strains [72].

**Fig. 10:**
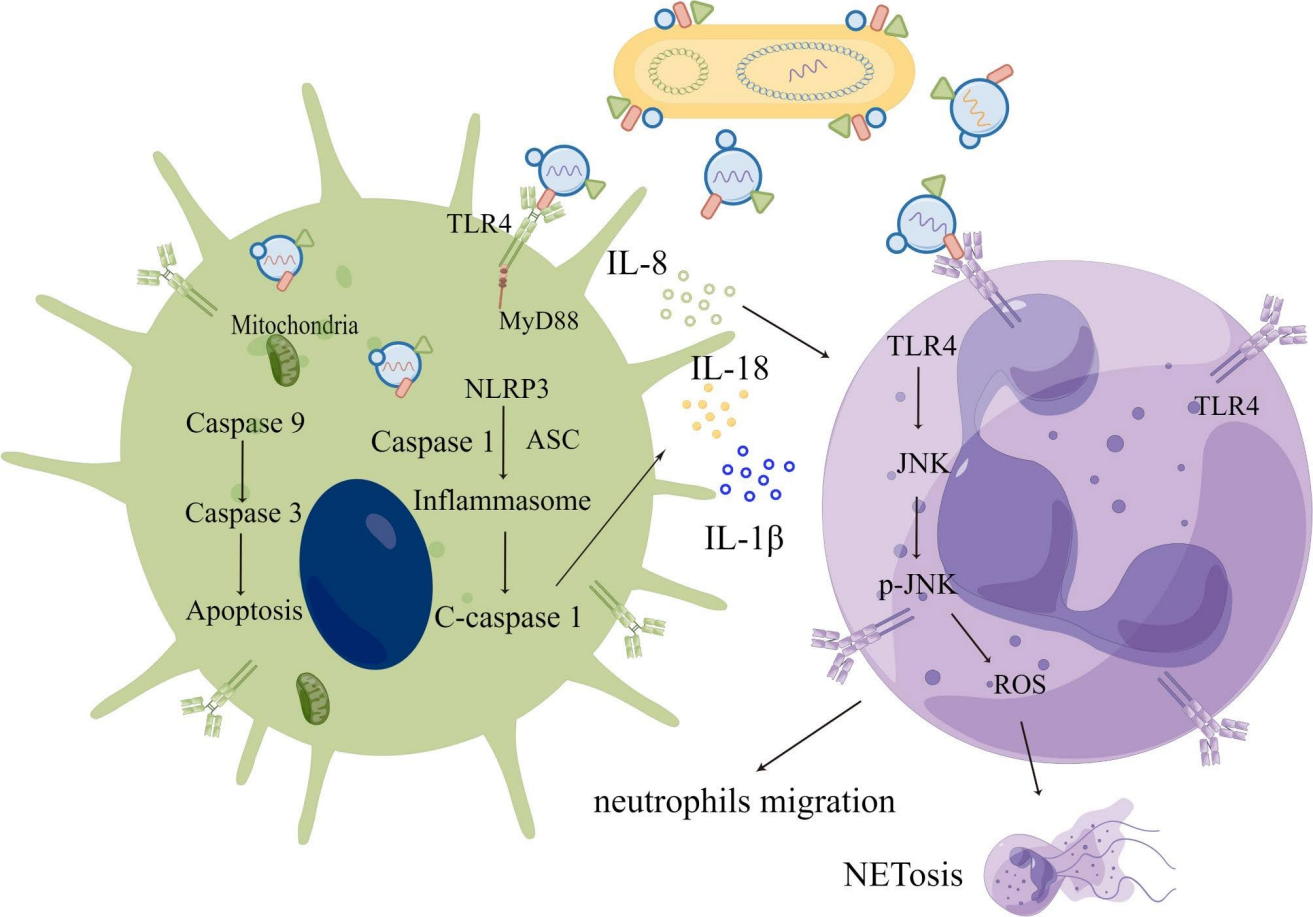
Schematic illustration of EVs interacting with immune cells. Intact EVs are released by APEC CT265 and target the immune cells. Both macrophages and neutrophils sense EVs through TLR4. EVs cause a proinflammatory response in macrophages by activating TLR4 and the NLRP3 inflammasome, triggering the release of proinflammatory cytokines and chemokines. Chemokines promote neutrophil infiltration. Cytosolic EVs also induce macrophage apoptosis via the mitochondria-dependent pathway. EVs induce neutrophils to release NETs via the SAPK/JNK signaling pathway, although the mechanism remains unclear

A previous study has shown that LPS usually induces inflammation and causes cell apoptosis [73]. Because EVs contain much LPS on their surfaces, we speculated that EVs from APEC CT265 would cause inflammation and apoptosis in macrophages. Intact EVs induced inflammation and apoptosis in HD11 and PMA-treated THP-1 macrophages. The EVs activated the TLR4 and NLRP3 signaling pathways and promoted the formation of the NLRP3 inflammasome, inducing the release of IL18, IL1β, and IL8 (Fig. 10). EVs also caused apoptosis in macrophages via the mitochondria-dependent pathway rather than the death receptor pathway. Previous studies have confirmed that EVs from Gram-negative bacteria activate intrinsic apoptosis [45, 46]. Neutrophils play an important role in antimicrobial defenses during infection. Chemokines promote neutrophil migration from the blood into specific tissues to destroy pathogenic microorganisms. EVs from APEC CT265 activated neutrophils to release NETs through the SAPK/JNK signaling pathway, inducing damage to the neutrophils, and ultimately allowing the pathogen to evade the immune response of the host (Fig. 10).

## Conclusion

Our research has demonstrated that APEC-derived EVs induced an inflammatory response in macrophages and NET formation in neutrophils, and that TLR4 was involved in the APEC-EV-activated inflammatory response. These findings provided a basis for the further study of APEC pathogenesis.

## Materials and methods

### Mice

Female BALB/C mice (4–6 weeks old) were purchased from the Comparative Medicine Center of Yangzhou University (Yangzhou, Jiangsu, China). TLR4 KO BALB/C mice were purchased from GemPharmatech. All animal experiments were conducted according to the regulations of experimental animal administration. The study was approved by the Ethical Committee for Animal Experiments of Nanjing Agricultural University, China (permit number: SYXK (Su) 2017–0007).

### Materials

RPMI-1640 medium, Dulbecco’s Modified Eagle’s Medium (DMEM), and fetal bovine serum (FBS) were purchased from Gibco (USA). Phorbol-12-myristate 13-acetate (PMA, P8139) and OptiPrep (D1556)were purchased from Sigma-Aldrich (Shanghai, China). Antibodies directed against NF-κB P-p65 (#9601), ASC (#67,824), caspase 1 (#3866), and NLRP3 (#13,158) were purchased from Cell Signaling Technology (CST, USA). Anti-myeloperoxidase (MPO, ab9535) antibody was purchased from Abcam (Cambridge, MA, USA). Antibodies directed against β-actin (81115-1-RR), NF-κB p65 (80979-1-RR), MYD88(23230-1-AP), TLR4(19811-1-AP), BAX(50599-2-Ig), BCL2(12789-1-AP), Caspase 3(19677-1-AP), Caspase 8(66093-1-Ig), Caspase 9(10380-1-AP) and horseradish peroxidase (HRP)-conjugated secondary antibodies(SA00001-2) were purchased from Proteintech (Wuhan, China). Goat anti-mouse fluorescein isothiocyanate (FITC) (16,852), Alex Fluor 647 (23,127) and Alex Fluor 555 (23,119) were purchased from AAT Bioquest (Sunnyvale, CA, USA). TAK-242 (a TLR4 inhibitor) was purchased from Selleck Chemicals (Selleck, Houston, TX, USA). SP600125 (an SAPK/JNK inhibitor) was purchased from Sigma-Aldrich (St. Louis, MO, USA). The mouse bone-marrow neutrophil isolation kit (TBD2013NM) and Chicken Heterophilic Isolation Kit (LZS1098C) were purchased from Tianjin Hao Yang Biological Products Technology Co., Ltd (Tianjin, China). The Whole Protein Extraction Kit and RNA Extraction Kit was purchased from TransGen Biotech Co., Ltd (Beijing, China). The BCA Protein Assay Kit was obtained from Thermo Fisher Scientific (23,225, Santa Clara, CA, USA).

### Bacterial culture and EV isolation

APEC strain CT265 was cultured in Luria-Bertani (LB) broth at 37 °C [74]. APEC mutant CT265Δ*wecA*, in which the *wecA* gene was deleted, was constructed as previously described [75]. To prepare the EVs, 20 mL of overnight bacterial culture was added to 1.0 L of LB broth supplemented with 1.0 mL of ampicillin (100 mg/mL). The bacteria were cultured in a thermostatic shaker (37 °C, 180 rpm; ZQZY-A8, Zhichu, Shanghai, China) for 12 h and then centrifuged (8,000 × *g*, 4 °C; Beckman, Germany) for 10 min. To remove any remaining bacteria, the supernatants were passed successively through sterile 0.45 and 0.22 μm filters. The supernatants were collected and ultracentrifuged by OptiPrep™ gradient ultracentrifugation with a 50.2 Ti rotor (200,000 × *g*, 4 °C, 2 h; Beckman). The precipitates were resuspended in 1.0 × TE buffer (pH 7.4), Each suspension was filtered through a 0.22 μm sterile filter and concentrated with an ultrafiltration tube (Millipore, Burlington, MA, USA). The precipitate was suspended in 500 µL of sterile TE buffer and stored at 4 °C.

### Determination of lipopolysaccharide (LPS) content in APEC EVs

The LPS content of the EVs was determined with the PyroGene™ Recombinant Factor C Endotoxin Detection Assay (Lonza, Basel, Switzerland), according to the manufacturer’s instructions, as previously reported [76]. Briefly, 100 µL of endotoxin standard, diluted EV sample, or a blank control were added to the appropriate wells of microplates and preincubated for 10 min at 37 °C. Then 100 µL of working reagent, consisting of recombinant factor C enzyme solution, fluorogenic substrate, and assay buffer, was added to each well. The optical density at 450 nm(OD450) of each well was measured with a fluorescence microplate reader, both immediately and after incubation for 1 h at 37 °C. The amount of LPS was calculated from a standard curve and the concentration was expressed as international units (IU) per mL. The experiments were repeated three times, and each sample was measured in triplicate.

### Termination of bacterial growth curves and EV kinetic curves

APEC strain CT265 was grown in 1.0 L of LB broth (37 °C, 180 rpm, 24 h) and 50 mL aliquots were collected at hourly intervals. The bacteria were removed by centrifugation (4 °C, 8,000× g, 3 h), and the supernatants were sterile filtered as described above. The EVs were isolated with ultracentrifugation (200,000 × *g*, 4 °C, 2 h), and the EV pellets were resuspended in 50 µL of 1.0 × TE buffer (pH 8.0). The EVs were then separated with SDS-PAGE and immunoblotted, using an anti-OmpA antibody to detect the protein bands. The signals were quantified densitometrically, as described above, with slight modification [77]. The results were recorded in arbitrary densitometric units (DU). Bacterial growth was monitored by measuring the OD at a wavelength of 600 nm (OD_600_) with an automatic biological growth monitoring reactor (Biosan, Riga, Latvia) at each time point.

### Cell culture

Human monocytes (THP-1 cells) were cultured in RPMI 1640 medium containing 10% FBS. Chicken macrophages (HD11 cell line) were cultured in DMEM with 10% FBS. The cells were maintained in a humidified incubator at 37 °C under 5% CO_2_. All Cells were plated in 25cm^2^ culture flasks (707,003, NEST, Wuxi, China).

### Isolation of mouse bone marrow neutrophils

Mouse bone marrow neutrophils were isolated with the mouse neutrophil isolation kit, according to the manufacturer’s instructions. Briefly, the femurs and tibiae were isolated from euthanized mice, and the bone-marrow cells were collected and passed through a 70 μm filter. The red blood cells were lysed. The samples were separated with density gradient centrifugation (400 ×g, 20 min). According to trypan blue exclusion, the viability of the neutrophils was > 90%. The isolated neutrophils were cultured in RPMI 1640 medium containing 10% FBS.

### Isolation of chicken heterophils

Twenty-eight-day-old white feather broilers were purchased from Nanjing Qinglongshan Animal Breeding Center (Nanjing city, China). All animal experiments were conducted according to the regulations of experimental animal administration. To evaluate whether APEC EVs induced the release of HETs from chicken heterophils. The heterophils were isolated with the Chicken Heterophilic Isolation Kit. Briefly, blood was collected from the wing veins of the chickens using a 22G needle with an angle of 30°, and collected in sodium-heparin tubes. The samples were separated with density gradient centrifugation (400 ×g, 20 min). The heterophil layer was collected and the red blood cells lysed. According to trypan blue exclusion, the viability of the heterophils was > 90%. The isolated heterophils were cultured in RPMI 1640 medium containing 10% serum.

### Transmission electron microscopy

Transmission electron microscopy (TEM) was used to observe the morphology of the bacteria and EVs. Briefly, APEC CT265 was cultured on LB agar plates (37 °C, 7 h) and then washed off the plates with ultrapure water (with resistivity 18 MΩ cm^− 1^, Millipore, France). The protein concentrations of the EVs were measured with the BCA Protein Assay Kit and adjusted to a concentration to 0.1 mg/mL. To prepare the TEM samples, 1.0 µL of bacterial liquid or EV solution was placed on a copper grid, then fixed and stained with 1% phosphotungstic acid aqueous solution for 90 s. Samples were analyzed at 80 kV on a Hitachi electron microscope and photographed (Hitachi, HT7800, Tokyo).

### Nanoparticle tracking analysis (NTA)

The size distributions and concentrations of the isolated EVs were determined with an NTA, as previously described [78]. EV solutions (50 µL) were collected and NTA was performed with a ZetaView® nanoparticle analyzer (Beijing ECHO Biotech, China).

### Cytotoxicity assays

The cytotoxicity of EVs to HD11 and THP-1 macrophages was determined with the CCK-8 assay, as previously reported [79]. Briefly, HD11 cells and PMA-differentiated THP-1 cells (5 × 10^5^/mL) were seeded in a 96-well plate and incubated with different concentrations of EVs (30, 40, or 50 µg/mL) for various periods (0, 0.5, 1.5, 4, 8, 16, and 24 h). Then, 10.0 µL of CCK-8 reagent was added to each well and incubated for 3 h at 37 °C, then the OD_450_ was measured.

### Immunofluorescence observation

To monitor the colocalization of EVs with HD11 and THP-1 cells over time, HD11 and PMA-treated THP-1 cells (5 × 10^5^/mL) were incubated with different concentrations of EVs (30, 40, or 50 µg/mL) in small confocal dishes for 12 h. The dishes were washed three times with phosphate-buffered saline (PBS), fixed with 4% paraformaldehyde, and permeabilized with 0.1% Triton X-100. After the EVs were blocked with 5% bovine serum albumin (BSA; Sigma-Aldrich), they were labeled with DiO (AAT Bioquest), according to the manufacturer’s instructions. Alexa-Fluor-647-conjugated phalloidin was used to stain F-actin and 4′,6-diamidino-2-phenylindole (DAPI) to stain the nuclei. The samples were washed with PBS, sealed with anti-fluorescence quenching sealing tablets (Biyuntian, China), and viewed under a laser scanning confocal microscope (Leica, Wetzlar, Germany).

To visualize the formation of NETs caused by the incubation of cells with EVs, mouse bone marrow neutrophils were seeded in small confocal dishes coated with polylysine (Sigma-Aldrich), and then incubated for 6 h with different doses of EVs labeled with DiO (50 µg/mL). The cells were then washed three times with PBS, fixed with 4% paraformaldehyde, and permeabilized with 0.1% Triton X-100. After the cells were blocked with 5% BSA, Alexa-Fluor-647-conjugated phalloidin (1.0 µL, dilution at 1:1000) was used to stain the F-actin and DAPI to stain the nuclei, and imaged with microscope (Axio Observer 7, Zess, Germany) at ×20 or ×40 magnification, the fluorescence intensity was 50%. For the control experiments, cells were incubated with EV buffer or 100 nM PMA for 6 h and treated as described above.

### RNA preparation, reverse transcription (RT), and quantitative real-time PCR

The total RNA from different types of cells was extracted with an RNA extraction kit (TransGen Biotech, Beijing, China), according to the manufacturer’s instructions. cDNA was generated with the HiScript II One-Step RT-PCR Kit (Vazyme Biotech, Nanjing, China), according to the manufacturer’s instructions. Quantitative real-time PCR (qPCR) was performed with SYBR Green qPCR Master Mix (Vazyme Biotech) and the StepOnePlus™ Real-Time PCR System (Applied Biosystems). The qPCR cycling parameters were: 95 °C for 5 min; 40 cycles of 95 °C for 10 s, 60 °C for 30 s; and final fluorescence detection at 60 °C for 1 min. The qPCR primers, designed with the National Center for Biotechnology Information (NCBI) Primer BLAST and synthesized by Tsingke (Nanjing, China), are shown in Table S1.

### Enzyme-linked immunosorbent assays (ELISAs)

The levels of cytokines secreted from macrophages were measured with ELISA kits (Dakewe), according to the manufacturer’s protocols. Briefly, THP-1 cells were seeded in a 96-well plate at a density of 5 × 10^5^/mL and incubated with EVs (50 µg/mL). After 12 h, the cell supernatants were collected, and the concentrations of interleukin 1β (IL1β), IL18, and tumor necrosis factor α (TNF-α) in the supernatants were measured. Briefly, the IL1β, IL18, and TNF-α antibodies were incubated in an ELISA plate for 1 h at 37 °C. The plate was washed with washing solution five times, then the secondary antibodies were added. Finally, the stop solution was added to the plate, and the values of OD450 were measured in each well within 10 min using a microplate reader. PBS served as a negative control.

### Western blotting

Western blotting was performed as described previously [80]. Briefly, cells were washed with cold PBS and treated with RIPA lysis buffer (TransGen Biotech). The cell lysates were mixed with SDS loading buffer and boiled for 10 min. The samples were subjected to SDS-PAGE and transferred onto polyvinylidene difluoride (PVDF) membrane (Bio-Rad, Hercules, CA, USA). The PVDF membrane was blocked with 5% skim milk in 1 × PBS at room temperature for 2 h. The membranes were incubated overnight at 4 °C with each primary antibody in blocking buffer, then washed three times with 0.5% PBS containing Tween 20 (PBST). An HRP-labeled goat anti-rabbit secondary antibody was added and incubated at 37 °C for 1 h, and the membrane was washed three times with 0.5‰ PBST. The signals of the target proteins were detected with ECL reagent (Vazyme Biotech). All assays in this experiment were performed independently three times, each as three replicates.

### Flow cytometry

The apoptosis of macrophages was detected with flow cytometry using an Annexin V-FITC/PI Apoptosis Detection Kit (Vazyme Biotech), according to the manufacturer’s instructions but with slight modifications [81]. Briefly, HD11 and PMA-treated THP-1 macrophages and were incubated with APEC EVs for 24 h. The cells were digested with EDTA-free trypsin. After collection, the samples were washed twice with cold PBS and resuspended in 100 µL of 1.0 × binding buffer. The cell suspensions were then incubated successively with 5.0 µL of annexin V-FITC and 5.0 µL of propidium iodide (PI) at room temperature for 15 min, protected from light. Cell apoptosis was analyzed with flow cytometry.

### Statistical analysis

The results are presented as the mean ± SEC for at least three independent experiments. Two group comparisons were done using Student’s t-test or paired t-test Differences among more than two groups were analyzed using one-way or two-way ANOVA. Values of *P* < 0.05 were considered statistically significant; *P* < 0.05 denoted by *, *P* < 0.01 denoted by **. The intensities of the protein bands were analyzed with the ImageJ software. Data obtained from the experiments were analyzed with the GraphPad Prism 7.0 software.

### Electronic supplementary material

Below is the link to the electronic supplementary material.

Supplementary Material 1: Figure 1(E) SDS-PAGE analysis of proteinase-K-treated EVs, untreated EVs, and CT265 whole-cell bacterial lysate (WC)

Supplementary Material 2: Figure 1(D) Western blotting analysis of EV protein OmpA after incubation for (2, 4, 6, 8, 10, 12, 14, 16, and 18 h). A representative western blot is shown

Supplementary Material 3: Figure 9 (A) Gating settings of flow cytometry

Supplementary Material 4: Table S1 Primers used for qPCR in the manuscript

## Data Availability

All other data are included within the article or are available upon request.

## Abbreviations

APEC: Avian Pathogenic *Escherichia coli*
NET: Neutrophil Extracellular Trap
EVs: Extracellular vesicles
LPS: Lipopolysaccharide
TLR4: Toll like receptor 4
ExPEC: Extraintestinal pathogenic *E. coli*
OMVs: Outer-membrane vesicles
OIMVs: Outer-inner membrane vesicles
EOMVs: Explosive outer membrane vesicles
CMVs: Cytoplasmic membrane vesicles
NE: Neutrophil elastase
MPO: Myeloperoxidase
PBS: Phosphate-buffered saline
TEM: Transmission electron microscopy
NTA: Nanoparticle tracking analysis
IL6: Interleukin 6
IL8: Interleukin 8
TNF-α: Tumor necrosis factorα
NCBI: National Center for Biotechnology Information
BSA: Bovine serum albumin
DAPI 4′: 6-diamidino-2-phenylindole
DMEM: Dulbecco’s Modified Eagle’s Medium
FBS: Fetal bovine serum
PMA: Phorbol-12-myristate 13-acetate
PRRs: Pattern-recognition receptors

## References

[1] Soleymani S, Tavassoli A, Hashemi Tabar G, Kalidari GA, Dehghani H (2020). Design, development, and evaluation of the efficacy of a nucleic acid-free version of a bacterial ghost candidate vaccine against avian pathogenic *E. coli* (APEC) O78:K80 serotype. Vet Res.

[2] Guabiraba R, Schouler C (2015). Avian colibacillosis: still many black holes. FEMS Microbiol Lett.

[3] Dho-Moulin M, Fairbrother JM (1999). Avian pathogenic *Escherichia coli* (APEC). Vet Res.

[4] Jeong J, Lee JY, Kang MS, Lee HJ, Kang SI, Lee OM, Kwon YK, Kim JH. Comparative characteristics and zoonotic potential of avian pathogenic *Escherichia coli* (APEC) isolates from chicken and duck in South Korea. Microorganisms 2021, 9. 10.3390/microorganisms9050946 PMC8145765 33925760

[5] Kathayat D, Lokesh D, Ranjit S, Rajashekara G. Avian pathogenic *Escherichia coli* (APEC): an overview of virulence and pathogenesis factors, zoonotic potential, and control strategies. Pathogens 2021, 10. 10.3390/pathogens10040467 PMC8069529 33921518

[6] Johnson JR, Kuskowski MA, Menard M, Gajewski A, Xercavins M, Garau J (2006). Similarity between human and chicken *Escherichia coli* isolates in relation to ciprofloxacin resistance status. J Infect Dis.

[7] Mellata M, Dho-Moulin M, Dozois CM, Curtiss R, Lehoux B, Fairbrother JM (2003). Role of avian pathogenic *Escherichia coli* virulence factors in bacterial interaction with chicken heterophils and macrophages. Infect Immun.

[8] Dziva F, Stevens MP (2008). Colibacillosis in poultry: unravelling the molecular basis of virulence of avian pathogenic *Escherichia coli* in their natural hosts. Avian Pathol.

[9] Reese S, Dalamani G, Kaspers B (2006). The avian lung-associated immune system: a review. Vet Res.

[10] Gao Q, Su S, Li X, Wang H, Liu J, Gao S (2020). Transcriptional analysis of RstA/RstB in avian pathogenic *Escherichia coli* identifies its role in the regulation of hded-mediated virulence and survival in chicken macrophages. Vet Microbiol.

[11] Zhuge X, Sun Y, Jiang M, Wang J, Tang F, Xue F, Ren J, Zhu W, Dai J (2019). Acetate metabolic requirement of avian pathogenic *Escherichia coli* promotes its intracellular proliferation within macrophage. Vet Res.

[12] Zhuge X, Sun Y, Xue F, Tang F, Ren J, Li D, Wang J, Jiang M, Dai J (2018). A novel PhoP/PhoQ regulation pathway modulates the survival of Extraintestinal pathogenic *Escherichia coli* in Macrophages. Front Immunol.

[13] Kaparakis-Liaskos M, Ferrero RL (2015). Immune modulation by bacterial outer membrane vesicles. Nat Rev Immunol.

[14] Toyofuku M, Nomura N, Eberl L (2019). Types and origins of bacterial membrane vesicles. Nat Rev Microbiol.

[15] Palomino RAN, Vanpouille C, Costantini PE, Margolis L. Microbiota-host communications: bacterial extracellular vesicles as a common language. PLoS Pathog 2021, 17. 10.1371/journal.ppat.1009508 PMC8118305 33984071

[16] Dhital S, Deo P, Stuart I, Naderer T (2021). Bacterial outer membrane vesicles and host cell death signaling. Trends Microbiol.

[17] Wang ZX, Wen Z, Jiang M, Xia FF, Wang M, Zhuge XK, Dai JJ. Dissemination of virulence and resistance genes among Klebsiella pneumoniae via outer membrane vesicle: an important plasmid transfer mechanism to promote the emergence of carbapenem-resistant hypervirulent *Klebsiella pneumoniae*. Transboundary and Emerging Diseases; 2022. 10.1111/tbed.14615 35679514

[18] Hu RJ, Li J, Zhao YZ, Lin H, Liang L, Wang MM, Liu HJ, Min YN, Gao YP, Yang MM. Exploiting bacterial outer membrane vesicles as a cross-protective vaccine candidate against avian pathogenic *Escherichia coli* (APEC). Microb Cell Fact 2020, 19. 10.1186/s12934-020-01372-7 PMC7268718 32493405

[19] Wang Y, Ke Y, Duan C, Ma X, Hao Q, Song L, Guo X, Sun T, Zhang W, Zhang J (2019). A small non-coding RNA facilitates *Brucella melitensis* intracellular survival by regulating the expression of virulence factor. Int J Med Microbiol.

[20] Li C, Wen RQ, Mu RR, Chen X, Ma P, Gu K, Huang ZR, Ju ZJ, Lei CW, Tang YZ, Wang HN. Outer membrane vesicles of avian pathogenic *Escherichia coli* mediate the horizontal transmission of bla(CTX-M-55). Pathogens 2022, 11. 10.3390/pathogens11040481 PMC9025603 35456156

[21] Jiang M, Wang Z, Xia F, Wen Z, Chen R, Zhu D, Wang M, Zhuge X, Dai J (2022). Reductions in bacterial viability stimulate the production of extra-intestinal pathogenic *Escherichia coli* (ExPEC) cytoplasm-carrying Extracellular vesicles (EVs). PLoS Pathog.

[22] Jung AL, Stoiber C, Herkt CE, Schulz C, Bertrams W, Schmeck B (2016). *Legionella pneumophila*-derived outer membrane vesicles promote bacterial replication in macrophages. PLoS Pathog.

[23] Bitto NJ, Cheng L, Johnston EL, Pathirana R, Phan TK, Poon IKH, O’Brien-Simpson NM, Hill AF, Stinear TP, Kaparakis-Liaskos M (2021). *Staphylococcus aureus* membrane vesicles contain immunostimulatory DNA, RNA and peptidoglycan that activate innate immune receptors and induce autophagy. J Extracell Vesicles.

[24] Balhuizen MD, van Dijk A, Jansen JWA, van de Lest CHA, Veldhuizen EJA, Haagsman HP (2021). Outer membrane vesicles protect Gram-Negative Bacteria against host defense peptides. mSphere.

[25] Vemula S, Shi JJ, Hanneman P, Wei L, Kapur R (2010). ROCK1 functions as a suppressor of inflammatory cell migration by regulating PTEN phosphorylation and stability. Blood.

[26] Tay H, Du Cheyne C, Demeyere K, De Craene J, De Bels L, Meyer E, Zijlstra A, Spiegelaere W. Depletion of embryonic Macrophages leads to a reduction in Angiogenesis in the Ex Ovo Chick Chorioallantoic membrane assay. Cells 2020, 10. 10.3390/cells10010005 PMC7822194 33375076

[27] Chazaud B (2014). Macrophages: supportive cells for tissue repair and regeneration. Immunobiology.

[28] Nikitina E, Larionova I, Choinzonov E, Kzhyshkowska J. Monocytes and macrophages as viral targets and Reservoirs. Int J Mol Sci 2018, 19. 10.3390/ijms19092821 PMC6163364 30231586

[29] Damascena HL, Silveira WAA, Castro MS, Fontes W. Neutrophil activated by the Famous and Potent PMA (Phorbol Myristate acetate). Cells 2022, 11. 10.3390/cells11182889 PMC9496763 36139464

[30] Halverson TWR, Wilton M, Poon KKH, Petri B, Lewenza S. DNA is an antimicrobial component of Neutrophil Extracellular Traps. PLoS Pathog 2015, 11. 10.1371/journal.ppat.1004593 PMC4295883 25590621

[31] Brinkmann V, Reichard U, Goosmann C, Fauler B, Uhlemann Y, Weiss DS, Weinrauch Y, Zychlinsky A (2004). Neutrophil extracellular traps kill bacteria. Science.

[32] Etulain J, Martinod K, Wong SL, Cifuni SM, Schattner M, Wagner DD (2015). P-selectin promotes neutrophil extracellular trap formation in mice. Blood.

[33] Kang L, Yu H, Yang X, Zhu Y, Bai X, Wang R, Cao Y, Xu H, Luo H, Lu L (2020). Neutrophil extracellular traps released by neutrophils impair revascularization and vascular remodeling after stroke. Nat Commun.

[34] Mehanny M, Koch M, Lehr CM, Fuhrmann G (2020). Streptococcal extracellular membrane vesicles are rapidly internalized by Immune cells and alter their cytokine release. Front Immunol.

[35] Du Teil Espina M, Fu Y, van der Horst D, Hirschfeld C, Lopez-Alvarez M, Mulder LM, Gscheider C, Haider Rubio A, Huitema M, Becher D et al. Coating and corruption of human neutrophils by bacterial outer membrane vesicles. Microbiol Spectr 2022:e0075322. 10.1128/spectrum.00753-22 PMC9602476 36000865

[36] Meganathan V, Moyana R, Natarajan K, Kujur W, Kusampudi S, Mulik S, Boggaram V (2020). Bacterial extracellular vesicles isolated from organic dust induce neutrophilic inflammation in the lung. Am J Physiol Lung Cell Mol Physiol.

[37] Nahui Palomino RA, Vanpouille C, Costantini PE, Margolis L (2021). Microbiota-host communications: bacterial extracellular vesicles as a common language. PLoS Pathog.

[38] Kim SY, Kim MH, Son JH, Kim SI, Yun SH, Kim K, Kim S, Shin M, Lee JC (2020). Outer membrane vesicles produced by Burkholderia cepacia cultured with subinhibitory concentrations of ceftazidime enhance pro-inflammatory responses. Virulence.

[39] Park HS, Back YW, Shin KW, Bae HS, Lee KI, Choi HG, Choi S, Lee HH, Choi CH, Park JK, Kim HJ (2019). *Mycobacterium tuberculosis* Rv3463 induces mycobactericidal activity in macrophages by enhancing phagolysosomal fusion and exhibits therapeutic potential. Sci Rep.

[40] Bauernfeind FG, Horvath G, Stutz A, Alnemri ES, MacDonald K, Speert D, Fernandes-Alnemri T, Wu J, Monks BG, Fitzgerald KA (2009). Cutting edge: NF-kappaB activating pattern recognition and cytokine receptors license NLRP3 inflammasome activation by regulating NLRP3 expression. J Immunol.

[41] Lehrer J, Vigeant KA, Tatar LD, Valvano MA (2007). Functional characterization and membrane topology of *Escherichia coli WecA*, a sugar-phosphate transferase initiating the biosynthesis of enterobacterial common antigen and O-antigen lipopolysaccharide. J Bacteriol.

[42] Smith AA, Corona-Torres R, Hewitt RE, Stevens MP, Grant AJ, Consortium GVV. Modification of avian pathogenic *Escherichia coli* chi 7122 lipopolysaccharide increases accessibility to glycoconjugate antigens. Microb Cell Fact 2022, 21. 10.1186/s12934-022-01903-4 PMC9449299 36071433

[43] Gupte R, Nandu T, Kraus WL (2021). Nuclear ADP-ribosylation drives IFNgamma-dependent STAT1alpha enhancer formation in macrophages. Nat Commun.

[44] Bielaszewska M, Ruter C, Bauwens A, Greune L, Jarosch KA, Steil D, Zhang W, He X, Lloubes R, Fruth A (2017). Host cell interactions of outer membrane vesicle-associated virulence factors of enterohemorrhagic *Escherichia coli* O157: intracellular delivery, trafficking and mechanisms of cell injury. PLoS Pathog.

[45] Deo P, Chow SH, Han ML, Speir M, Huang C, Schittenhelm RB, Dhital S, Emery J, Li J, Kile BT (2020). Mitochondrial dysfunction caused by outer membrane vesicles from Gram-negative bacteria activates intrinsic apoptosis and inflammation. Nat Microbiol.

[46] Bielaszewska M, Ruter C, Kunsmann L, Greune L, Bauwens A, Zhang W, Kuczius T, Kim KS, Mellmann A, Schmidt MA, Karch H (2013). Enterohemorrhagic *Escherichia coli* hemolysin employs outer membrane vesicles to target mitochondria and cause endothelial and epithelial apoptosis. PLoS Pathog.

[47] Kunsmann L, Ruter C, Bauwens A, Greune L, Gluder M, Kemper B, Fruth A, Wai SN, He X, Lloubes R (2015). Virulence from vesicles: novel mechanisms of host cell injury by *Escherichia coli* O104:H4 outbreak strain. Sci Rep.

[48] Johar A, Al-Thani N, Al-Hadidi SH, Dlissi E, Mahmoud MH, Eltai NO. Antibiotic resistance and virulence gene patterns Associated with Avian Pathogenic *Escherichia coli* (APEC) from broiler chickens in Qatar. Antibiot (Basel) 2021, 10. 10.3390/antibiotics10050564 PMC8151107 34064966

[49] Subedi M, Luitel H, Devkota B, Bhattarai RK, Phuyal S, Panthi P, Shrestha A, Chaudhary DK (2018). Antibiotic resistance pattern and virulence genes content in avian pathogenic *Escherichia coli* (APEC) from broiler chickens in Chitwan, Nepal. BMC Vet Res.

[50] Palaniyandi S, Mitra A, Herren CD, Zhu X, Mukhopadhyay S (2013). LuxS contributes to virulence in avian pathogenic *Escherichia coli* O78:K80:H9. Vet Microbiol.

[51] Li Q, Yin L, Xue M, Wang Z, Song X, Shao Y, Liu H, Tu J, Qi K (2020). The transcriptional regulator PhoP mediates the tolC molecular mechanism on APEC biofilm formation and pathogenicity. Avian Pathol.

[52] Zhuge X, Tang F, Zhu H, Mao X, Wang S, Wu Z, Lu C, Dai J, Fan H (2016). AutA and AutR, two Novel Global Transcriptional regulators, facilitate avian pathogenic *Escherichia coli* infection. Sci Rep.

[53] Canas MA, Fabrega MJ, Gimenez R, Badia J, Baldoma L (2018). Outer membrane vesicles from probiotic and commensal *Escherichia coli* activate NOD1-Mediated Immune responses in intestinal epithelial cells. Front Microbiol.

[54] Bielaszewska M, Marejkova M, Bauwens A, Kunsmann-Prokscha L, Mellmann A, Karch H (2018). Enterohemorrhagic *Escherichia coli* O157 outer membrane vesicles induce interleukin 8 production in human intestinal epithelial cells by signaling via toll-like receptors TLR4 and TLR5 and activation of the nuclear factor NF-kappaB. Int J Med Microbiol.

[55] Li C, Wen R, Mu R, Chen X, Ma P, Gu K, Huang Z, Ju Z, Lei C, Tang Y, Wang H. Outer Membrane Vesicles of Avian Pathogenic *Escherichia coli* Mediate the Horizontal Transmission of blaCTX-M-55. *Pathogens* 2022, 11. 10.3390/pathogens11040481 PMC9025603 35456156

[56] Mellata M, Ameiss K, Mo H, Curtiss R (2010). 3rd: characterization of the contribution to virulence of three large plasmids of avian pathogenic *Escherichia coli* chi7122 (O78:K80:H9). Infect Immun.

[57] Wang J, Li F, Sun R, Gao X, Wei H, Li LJ, Tian Z (2013). Bacterial colonization dampens influenza-mediated acute lung injury via induction of M2 alveolar macrophages. Nat Commun.

[58] Klein JC, Moses K, Zelinskyy G, Sody S, Buer J, Lang S, Helfrich I, Dittmer U, Kirschning CJ, Brandau S (2017). Combined toll-like receptor 3/7/9 deficiency on host cells results in T-cell-dependent control of tumour growth. Nat Commun.

[59] O’Neill LAJ, Bowie AG (2007). The family of five: TIR-domain-containing adaptors in toll-like receptor signalling. Nat Rev Immunol.

[60] Wang Q, Lin P, Li P, Feng L, Ren Q, Xie X, Xu J (2017). Ghrelin protects the heart against ischemia/reperfusion injury via inhibition of TLR4/NLRP3 inflammasome pathway. Life Sci.

[61] Lee MS, Min YJ (2007). Signaling pathways downstream of pattern-recognition receptors and their cross talk. Annu Rev Biochem.

[62] Mocsai A, Walzog B, Lowell CA (2015). Intracellular signalling during neutrophil recruitment. Cardiovascular Res.

[63] Thomas CJ, Schroder K (2013). Pattern recognition receptor function in neutrophils. Trends Immunol.

[64] Swain DK, Kushwah MS, Kaur M, Patbandha TK, Mohanty AK, Dang AK (2014). Formation of NET, phagocytic activity, surface architecture, apoptosis and expression of toll like receptors 2 and 4 (TLR2 and TLR4) in neutrophils of mastitic cows. Vet Res Commun.

[65] Rizzo A, Losacco A, Carratelli CR, Di Domenico M, Bevilacqua N (2013). Lactobacillus plantarum reduces *Streptococcus pyogenes* virulence by modulating the IL-17, IL-23 and toll-like receptor 2/4 expressions in human epithelial cells. Int Immunopharmacol.

[66] Khan MA, Farahvash A, Douda DN, Licht JC, Grasemann H, Sweezey N, Palaniyar N. JNK activation turns on LPS- and gram-negative Bacteria-Induced NADPH oxidase-dependent suicidal NETosis. Sci Rep 2017, 7. 10.1038/s41598-017-03257-z PMC5469795 28611461

[67] Hakkim A, Fuchs TA, Martinez NE, Hess S, Prinz H, Zychlinsky A, Waldmann H (2011). Activation of the Raf-MEK-ERK pathway is required for neutrophil extracellular trap formation. Nat Chem Biol.

[68] Keshari RS, Verma A, Barthwal MK, Dikshit M (2013). Reactive oxygen species-induced activation of ERK and p38 MAPK mediates PMA-induced NETs release from human neutrophils. J Cell Biochem.

[69] Tamassia N, Calzetti F, Ear T, Cloutier A, Gasperini S, Bazzoni F, McDonald PP, Cassatella MA (2007). Molecular mechanisms underlying the synergistic induction of CXCL10 by LPS and IFN-gamma in human neutrophils. Eur J Immunol.

[70] Haeusgen W, Herdegen T, Waetzig V (2011). The bottleneck of JNK signaling: Molecular and functional characteristics of MKK4 and MKK7. Eur J Cell Biol.

[71] Sahr T, Escoll P, Rusniok C, Bui S, Pehau-Arnaudet G, Lavieu G, Buchrieser C (2022). Translocated *Legionella pneumophila* small RNAs mimic eukaryotic microRNAs targeting the host immune response. Nat Commun.

[72] Xia F, Jiang M, Wen Z, Wang Z, Wang M, Xu Y, Zhuge X, Dai J (2022). Complete genomic analysis of ST117 lineage extraintestinal pathogenic *Escherichia coli* (ExPEC) to reveal multiple genetic determinants to drive its global transmission: ST117 E. coli as an emerging multidrug-resistant foodborne ExPEC with zoonotic potential. Transbound Emerg Dis.

[73] Zhang D, Cao X, Li J, Zhao G (2015). MiR-210 inhibits NF-kappaB signaling pathway by targeting DR6 in osteoarthritis. Sci Rep.

[74] Meng X, Chen Y, Wang P, He M, Shi Y, Lai Y, Zhu G, Wang H. RyhB in avian pathogenic *Escherichia coli* regulates the expression of virulence-related genes and contributes to Meningitis Development in a mouse model. Int J Mol Sci 2022, 23. 10.3390/ijms232415532 PMC9778962 36555174

[75] Smith AA, Corona-Torres R, Hewitt RE, Stevens MP, Grant AJ (2022). Glycoengineering of Veterinary Vaccines C: modification of avian pathogenic *Escherichia coli* chi7122 lipopolysaccharide increases accessibility to glycoconjugate antigens. Microb Cell Fact.

[76] Liu KS, Zhang C, Dong HL, Li KK, Han QB, Wan Y, Chen R, Yang F, Li HL, Ko CH, Han XQ (2019). GSP-2, a polysaccharide extracted from Ganoderma sinense, is a novel toll-like receptor 4 agonist. PLoS ONE.

[77] Bielaszewska M, Ruter C, Kunsmann L, Greune L, Bauwens A, Zhang WL, Kuczius T, Kim KS, Mellmann A, Schmidt MA, Karch H. Enterohemorrhagic *Escherichia coli* Hemolysin employs outer membrane vesicles to Target Mitochondria and cause endothelial and epithelial apoptosis. PLoS Pathog 2013, 9. 10.1371/journal.ppat.1003797 PMC3861543 24348251

[78] Connolly KD, Guschina IA, Yeung V, Clayton A, Draman MS, Von Ruhland C, Ludgate M, James PE, Rees DA (2015). Characterisation of adipocyte-derived extracellular vesicles released pre- and post-adipogenesis. J Extracell Vesicles.

[79] Miller-Ocuin JL, Liang X, Boone BA, Doerfler WR, Singhi AD, Tang D, Kang R, Lotze MT, Zeh HJ (2019). 3rd: DNA released from neutrophil extracellular traps (NETs) activates pancreatic stellate cells and enhances pancreatic tumor growth. Oncoimmunology.

[80] Rong D, Lu C, Zhang B, Fu K, Zhao S, Tang W, Cao H (2019). CircPSMC3 suppresses the proliferation and metastasis of gastric cancer by acting as a competitive endogenous RNA through sponging miR-296-5p. Mol Cancer.

[81] Wang Z, Liu B, Ma X, Wang Y, Han W, Xiang L (2022). lncRNA ZFAS1 promotes intervertebral disc degeneration by upregulating AAK1. Open Med (Wars).

