# Supplementary figures and images for: Extracellular vesicles produced by avian pathogenic Escherichia coli (APEC) activate macrophage proinflammatory response and neutrophil extracellular trap (NET) formation through TLR4 signaling
Source: Microb Cell Fact. 2023 Sep 9;22:177. doi: 10.1186/s12934-023-02171-6 (PMC10492386; doi:10.1186/s12934-023-02171-6)

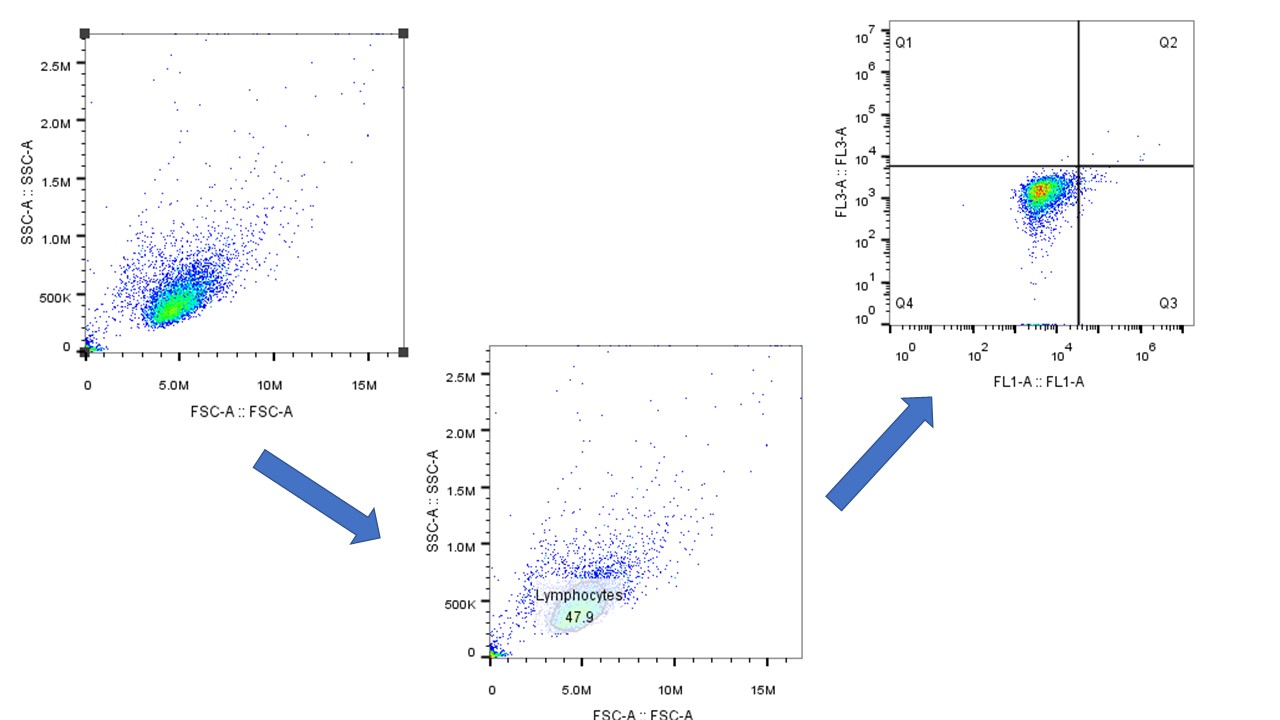

Supplement: Supplementary file 1 — Supplementary Material 1: Figure 1(E) SDS-PAGE analysis of proteinase-K-treated EVs, untreated EVs, and CT265 whole-cell bacterial lysate (WC) [file 12934_2023_2171_MOESM1_ESM.jpg]

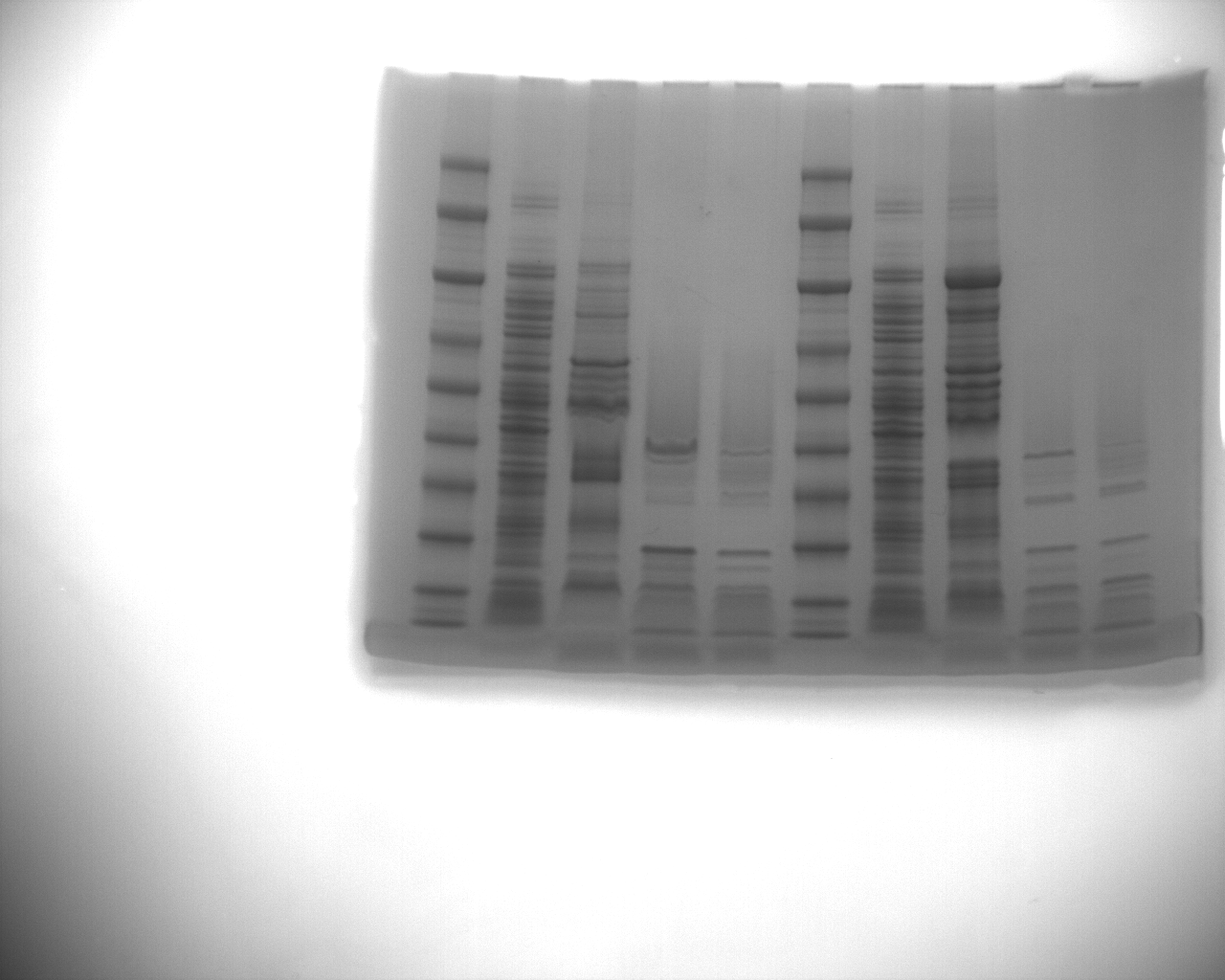

Supplement: Supplementary file 2 — Supplementary Material 2: Figure 1(D) Western blotting analysis of EV protein OmpA after incubation for (2, 4, 6, 8, 10, 12, 14, 16, and 18 h). A representative western blot is shown [file 12934_2023_2171_MOESM2_ESM.png]

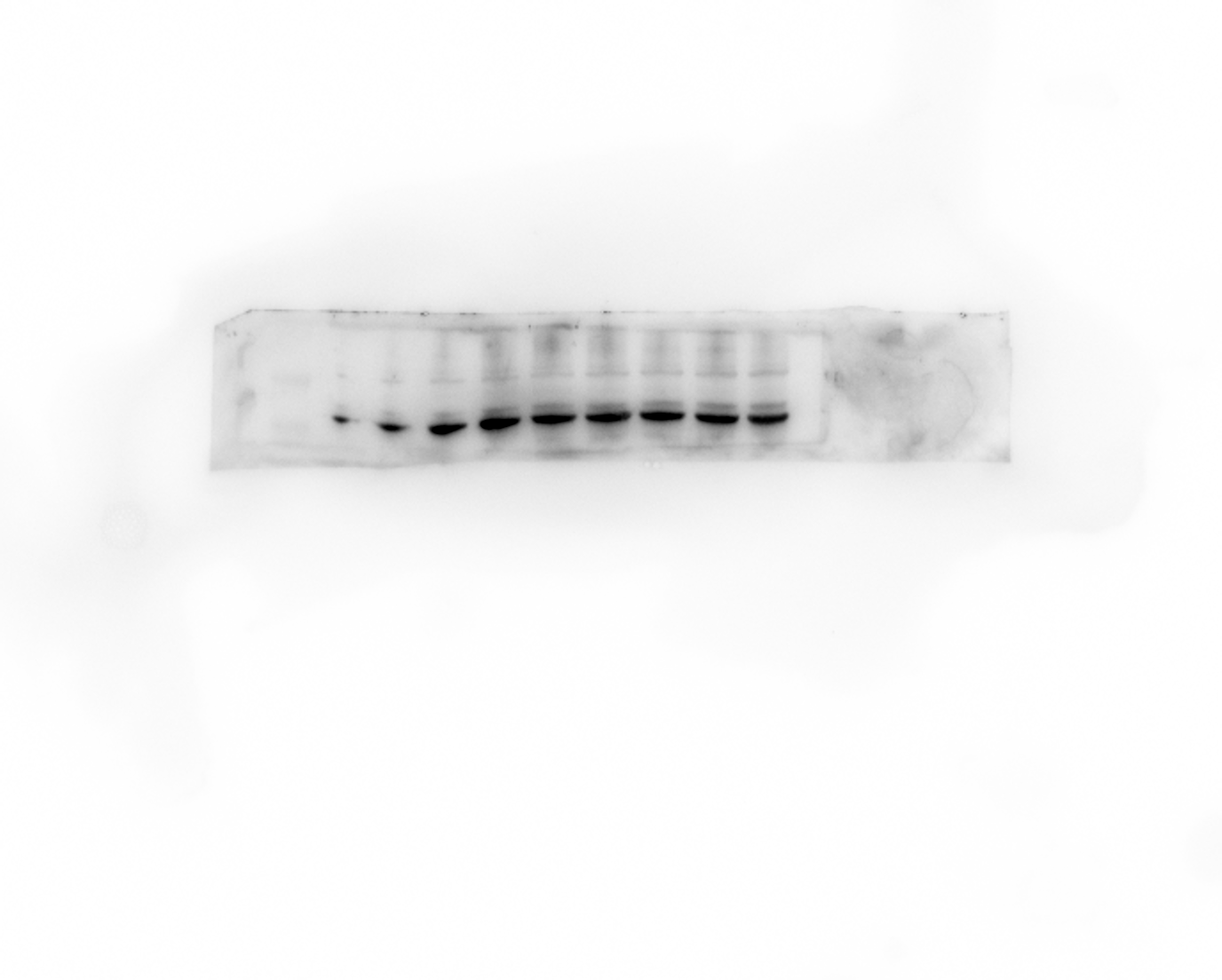

Supplement: Supplementary file 3 — Supplementary Material 3: Figure 9 (A) Gating settings of flow cytometry [file 12934_2023_2171_MOESM3_ESM.png]
