# Supplementary material for: Extracellular vesicles produced by avian pathogenic Escherichia coli (APEC) activate macrophage proinflammatory response and neutrophil extracellular trap (NET) formation through TLR4 signaling
Source: Microb Cell Fact. 2023 Sep 9;22:177. doi: 10.1186/s12934-023-02171-6 (PMC10492386; doi:10.1186/s12934-023-02171-6)
Supplement: Supplementary file 4 — Supplementary Material 4: Table S1 Primers used for qPCR in the manuscript [file 12934_2023_2171_MOESM4_ESM.docx]

**1. Primers used for qPCR**

| Gene Name Primer (5'-3') Accession Numbers |
| --- |
| **THP-1**  TLR4 F: CCCTGCGTGGAGACTTGG NM_003266.4  R: AGCAATGGAATCGGGGTGAA  MYD88 F: GCATATGCCTGAGCGTTTCG NM_001374787.1  R: ATCCGGCGGCACCCAC  GAPDH F: CACTAGGCGCTCACTGTTCT NM_001289746.2  R: GCCCAATACGACCAAATCCGT  **HD11**  TLR4 F: TGGATCTTTCAAGGTGCCACA KP410249.1  R: AGTGTCCGATGGGTAGGTCA  MYD88 F: AGGGATGATCCGTATGGGCA NM_001030962.5  R: ACACGTTCCTGGCAAGACAT  GAPDH F: TCGGAGTCAACGGATTTGGC NM_204305.2  R: ACAGTGCCCTTGAAGTGTCC |
